# Reversing T Cell Dysfunction to Boost Glioblastoma Immunotherapy by Paroxetine‐Mediated GRK2 Inhibition and Blockade of Multiple Checkpoints through Biomimetic Nanoparticles

**DOI:** 10.1002/advs.202204961

**Published:** 2023-01-25

**Authors:** Tingting Wang, Hao Zhang, Yaobao Han, Qing Zheng, Hanghang Liu, Mengxiao Han, Zhen Li

**Affiliations:** ^1^ Center for Molecular Imaging and Nuclear Medicine State Key Laboratory of Radiation Medicine and Protection School for Radiological and Interdisciplinary Sciences (RAD‐X) Suzhou Medical College of Soochow University Collaborative Innovation Center of Radiation Medicine of Jiangsu Higher Education Institutions Suzhou 215123 P. R. China

**Keywords:** biomimetic nanoparticles, glioblastoma, immunotherapy, old drug for new application, T cell dysfunction

## Abstract

T cell dysfunction‐induced tumor immune escape is particularly severe in glioblastoma (GBM), and significantly affects the efficacy of immunotherapy. It is crucial to innovatively reverse the T cell dysfunction for improving GBM immunotherapy. Herein, T cell dysfunction is remarkably reversed and immunotherapy of GBM is boosted by repurposing the U. S. Food and Drug Administration‐approved antidepressant paroxetine (PX) with biomimetic nanoparticles (CS‐J@CM/6 NPs). The PX is successfully applied to abrogate T cell sequestration in the bone marrow of GBM‐bearing mice and increase their infiltration in tumor. The biomimetic NPs are composed of ultrasmall Cu_2−_ *_x_* Se NPs, JQ1, and tumor cell membrane modified with CD6, and are efficiently delivered into tumor through the specific interactions between CD6 and activated leukocyte cell adhesion molecule. They ameliorate the T cell dysfunction through the double roles of loaded JQ1, which simultaneously decreases the expression of PD‐1 and TIM‐3 on T cells, and the expression of PD‐L1 on tumor cells. The NP also induces the immunogenic cell death of tumor cells to activate immune response. The synergistic roles of PX and biomimetic CS‐J@CM/6 NPs notably enhance the survival of GBM‐bearing mice. This work provides new insights into tumor immunotherapy by repurposing “old drugs” with advanced NPs.

## Graphical abstract

Reversing T cell dysfunction to significantly boost glioblastoma immunotherapy is achieved by repurposing “old drug” paroxetine (PX) with Cu_2−_ *_x_* Se‐based biomimetic nanoparticles. PX can release the sequestrated T cells in the bone marrow to increase their amounts and infiltration in tumor. Biomimetic nanoparticles can simultaneously reduce the expressions of immune checkpoints on tumor cells and T cells.

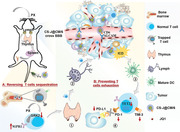

## 1 Introduction

High‐grade invasive glioblastoma (GBM) has a very poor prognosis less than 5% of 5 years survival rate worldwide.^[^ ^1^ ^]^ The gold‐standard treatment of GBM is the combination of the maximal surgical resection with temozolomide chemotherapy, and adjuvant radiotherapy.^[^ ^2^, ^3^ ^]^ Recently, activating the immune response of host to suppress cancer cells has brought revolutionary breakthroughs in the nonsmall cell lung cancer and melanoma cancer.^[^ ^4^, ^5^ ^]^ Unfortunately, the progress in the GBM immunotherapy is very slow because of the disappointed failure from the phase III clinical trial with anti‐PD‐1 therapy.^[^ ^6^, ^7^ ^]^ The failure was mainly attributed to the severe tumor immune escape in GBM, which was caused by the decrease of T cells and their dysfunction.^[^ ^8^, ^9^, ^10^, ^11^, ^12^, ^13^ ^]^ Therefore, it is of great importance to increase the infiltration of T cells into tumor and improve their functions for achieving excellent immunotherapy efficacy.

Lymphopenia in the GBM patients is prominently contributed to the depletion of T cells, leading to the failure of GBM immunotherapy. The reason of less infiltrated T cells in GBM was revealed by Fecci et al. They found that the growth of tumor could cause the loss of surface sphingosine‐1‐phosphate receptor 1 (S1PR1) on T cells, giving rise to the sequestration of T cells in the bone marrow (BM).^[^ ^14^ ^]^ S1PR1 is one of the five G protein‐coupled receptors that bind the second messenger sphingosine‐1‐phosphate (S1P).^[^ ^15^, ^16^ ^]^ The S1P‐S1PR1 axis serves as a switch of naïve T cells egressing from thymus and the secondary lymphoid organs.^[^ ^17^, ^18^ ^]^ When the S1PR1 on the naïve T cells is reduced, a large numbers of T cells are sequestered in the BM, leading to the deficiency of T cells in the blood and lymphoid organs. Therefore, hindering S1PR1 internalization and reversing sequestration of T cells for activating immunotherapy is sorely needed. Fecci et al. used the granulocyte colony‐stimulating factor to release the sequestered T cells in the BM, and improved ≈40% survival by jointly using it with 4‐1BB agonist.^[^ ^14^ ^]^

In addition to the amounts of infiltrated T cells in tumor, the T cell dysfunction can also lead to the failure of GBM immunotherapy. This is because the persistent antigen stimulation at tumor site can exhaust the T cells to be PD‐1^+^TIM‐3^+^ T cells.^[^ ^19^, ^20^, ^21^, ^22^, ^23^ ^]^ T cell exhaustion represents a specific transcriptional program in T cells, which is often characterized by the increased expression of multiple co‐inhibitory receptors, many of them constitute traditional and novel immune checkpoints, such as the programmed cell death 1 (PD‐1), T‐cell immunoglobulin and mucin‐domain‐containing molecule‐3 (TIM‐3), cytotoxic T‐lymphocyte antigen‐4 (CTLA‐4), and so on.^[^ ^24^ ^]^ Blocking these checkpoints can reduce the exhaustion of T cells and improve the therapeutic efficacy. However, the blockade of immune checkpoints with single monoclonal antibody has a very limited therapeutic efficacy in the GBM. An important reason is the difficulty in efficiently delivering large‐sized monoclonal antibodies into tumor because of the blood–brain barrier (BBB) issue, leading to their unsatisfactory therapeutic effects on the GBM. Furthermore, only blocking one type of immune checkpoints does not work well. It is crucial to prevent the T cell exhaustion through blockade of multiple immune checkpoints for improving the efficacy of tumor immunotherapy.^[^ ^25^, ^26^ ^]^ For example, Hung et al. used anti‐galectin‐9 (Gal‐9) antibody and agonistic antibody (DTA‐1) to co‐stimulate GITR (glucocorticoid‐induced tumor necrosis factor receptor‐related protein) receptor to reduce the expression of PD‐1 and TIM‐3 on T cells and enhance immunotherapy.^[^ ^27^ ^]^

The use of co‐inhibitory receptors can achieve a better efficacy of immunotherapy. However, the antibodies for different receptors hold different pharmacokinetics, immune‐related toxicities, and tumor penetration.^[^ ^28^, ^29^ ^]^ They also face the issue of crossing the BBB. It is highly desired to use the same ligand for targeting multiple different receptors. In this context, some small molecule ligands have attracted considerable interest.^[^ ^30^ ^]^ For example, Fraietta et al. used the potent and selective extra‐terminal bromodomain and extraterminal domain (BET) small molecular inhibitor JQ1 to downregulate the tet methylcytosine dioxygenase 2 (TET2) expression for simultaneously decreasing the expression of PD‐1 and TIM‐3 on T cells. JQ1 is a major epigenetic regulator of T cell differentiation, proliferation, and sustainable antitumor function for chimeric antigen receptor T therapy.^[^ ^31^ ^]^

The above examples demonstrate the importance of simultaneous increase of the infiltrated T cells and prevention of their exhaustion in tumor for improving the immunotherapy of GBM. As S1PR1 is endocytosed through G protein‐coupled receptor (GPCR) kinase 2 (GRK2)‐dependent phosphorylation.^[^ ^32^ ^]^ Inhibiting GRK2‐mediated GPCR desensitization could stabilize S1PR1 on T cell surface. Although several GRK2 inhibitors have been successfully tested in research, none of them has been approved by the U. S. Food and Drug Administration (FDA) for clinical practice. It is of great importance to repurpose FDA approved agents as GRK2 inhibitors. Tesmer et al. found that paroxetine (PX) as a GRK2 inhibitor functioned well both in vitro and in vivo.^[^ ^33^ ^]^ Kamal et al. used PX as a GRK2‐inhibitor to suppress the GPCR desensitization for treatment of osteoarthritis.^[^ ^34^ ^]^ These examples suggest that low toxic “old drug” PX could serve as a GRK2 inhibitor to stabilize S1PR1 on T cell surface for releasing the sequestered T cells in the BM to boost GBM immunotherapy, which has not been investigated and reported.

**Scheme 1:**
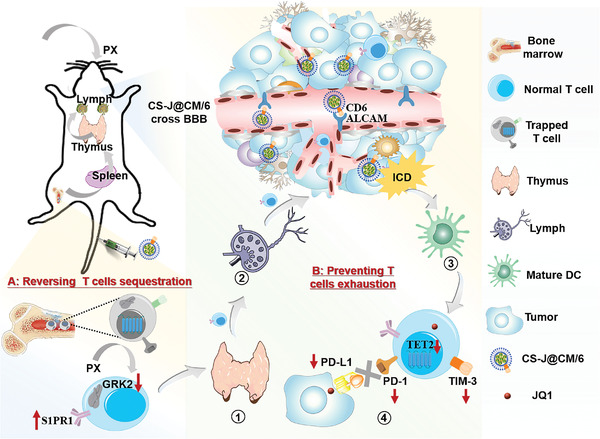
Schematic illustration of abrogation of T cell sequestration in the BM by “old drug” PX, and amelioration of T cell dysfunction by CS‐J@CM/6 NPs with capability of crossing the BBB through the strong interaction between CD6 and ALCAM expressed on the microvascular endothelium of the BBB. 1) T cells egress from BM to thymus. 2) T cells egress from thymus to lymph node. 3) ROS generated by CS‐J@CM/6 NPs induce the immunogenic cell death of GL261 cells to release damage‐associated molecular patterns to activate dendritic cells (DCs) for maturation. 4) PD‐1 and TIM‐3 on T cells, and PD‐L1 on tumor cells are decreased by CS‐J@CM/6 NPs to ameliorate T cell dysfunction caused by GBM.

In this article, we report the increased infiltration of T cells and improvement of T cell dysfunction to boost the GBM immunotherapy by jointly using biomimetic nanoparticles (termed as CS‐J@CM/6 NPs) and PX (**Scheme** **1**). In our rational design, the FDA‐approved “old drug” PX was first used as a GRK2 inhibitor to increase the S1PR1 expression on T cells. Oral administration of PX into GBM‐bearing mice freed their sequestered T cells in the BM to increase the amount and infiltration of T cells in the tumor. Second, JQ1 as a BET molecule was loaded onto ultrasmall Cu_2−_ *_x_* Se nanoparticles (the resultant nanoparticles were abbreviated as CS‐J NPs), and used to decrease not only PD‐1 and TIM‐3 on T cells through downregulation of TET2 expression, but also PD‐L1 on tumor cells. The simultaneous decrease of multiple receptors synergetically improved T cell function and activity. Third, CS‐J NPs were coated with tumor cell membrane and functionalized with CD6 to facilitate them crossing the BBB through the specific interactions between CD6 and activated leukocyte cell adhesion molecule (ALCAM) expressed by endothelium, and then targeting tumor cells by the homologous adhesion effect.^[^ ^35^ ^]^ Lastly, Cu_2−_ *_x_* Se NPs from the CS‐J@CM/6 NPs generated reactive oxygen species (ROS) through their Fenton‐like reaction to activate the immune response due to the release of tumor‐associated antigen from the immunogenic cell death (ICD) of tumor cells.^[^ ^36^, ^37^ ^]^ Overall, this work demonstrates the great potential of combination of our biomimetic nanoparticles with “old drug” PX in boosting GBM immunotherapy through simultaneous increase of infiltrated T cells and improvement of their dysfunction via multiple effects.

## 2 Results and Discussion

**Figure 1:**
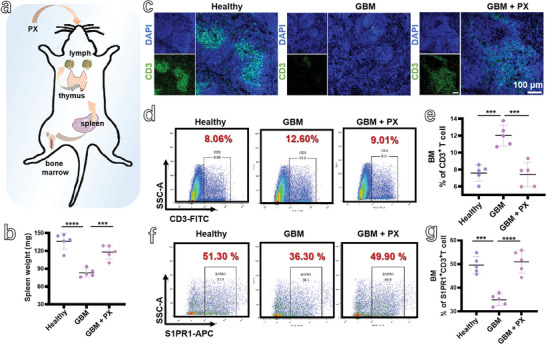
a) Schematic illustration of PX releasing T cells sequestrated in the BM to increase their infiltration in tumor. b) The spleen weights of healthy mice, GBM‐bearing mice, and the GBM‐bearing mice treated with PX (*n* = 5). c) Immunofluorescence images of CD3^+^ T cells in the spleen of mice from different groups (scale bar: 100 µm). d,e) Flow cytometry analysis of CD3^+^ T cells and their percentage in the BM of mice from Healthy group, GBM group, and GBM + PX group (*n* = 5). f,g) Flow cytometry analysis of S1PR1^+^CD3^+^ T cells and their percentage in the BM of mice from different groups (*n* = 5).

It is known that GBM patients show less T cells (including CD8^+^ T cells and CD4^+^ T cells) in their spleen, because of the T cell lymphopenia caused by the loss of S1PR1 on T cells. The S1PR1 is one of the five G protein‐coupled receptors bonding with the sphingosine‐1‐phosphate (S1P),^[^ ^15^, ^38^ ^]^ and the S1P‐S1PR1 axis controls the trafficking of lymphocytes. To assess the change of T cells and their surface S1PR1 in the GBM‐bearing mice, GL261 cells were stereotactically implanted into the brain of C57BL/6 mice to create the GBM model. The BM, spleen, blood, and cervical lymph nodes (CLNs) of tumor‐bearing mice were harvested for analysis after they were treated by oral administration of PX (**Figure** **1**a). To our surprise, the spleen of all GBM‐bearing mice was shrunk (Figure S1, Supporting Information). It was recovered to some extent after treatment with PX for four times (10 mg kg^−1^ every other day). As shown in Figure 1b, the average weight of spleen in the GBM‐bearing mice was only 60% that of healthy mice. The spleen weight of the GBM‐bearing mice treated with PX was recovered to around 87% that of healthy mice.

As spleen is the typical immune organ, T cells in the spleen of healthy mice, GBM‐bearing mice, and the GBM‐bearing mice treated with PX were analyzed by immunofluorescence. A bright green fluorescence of CD3^+^ T cells from the spleen of GBM‐bearing mice treated with PX was observed (Figure 1c), which was similar to the spleen of healthy mice. In contrast, the immunofluorescence in the spleen of GBM‐bearing mice was very weak, suggesting very few CD3^+^ T cells. Apart from the spleen, T cells in the BM were also detected. Figure 1d,e shows that significant amounts of T cells were sequestrated in the BM of GBM‐bearing mice. The CD3^+^ T cells in the BM of GBM‐bearing mice were 12.60%, which was much higher and around 1.5‐fold of that from healthy mice (8.06%). However, after the GBM‐bearing mice were treated with PX, their CD3^+^ T cells were decreased to 9.01%, which was almost the same as that of healthy mice. The S1PR1^+^CD3^+^ T cells were also detected and are shown in Figure 1f,g. The decreased S1PR1^+^CD3^+^ T cells were obviously observed in the GBM‐bearing mice, which were recovered to the similar level of healthy mice after the tumor‐bearing mice were treated with PX. The variation of CD8^+^ T cells in the BM of mice (Figure S2, Supporting Information) was similar to the CD3^+^ T cells. These results illustrate that the PX can increase the S1PR1 expression to afford T cells egressing from the BM to the lymphokinesis.

In addition to the BM, the changes of CD3^+^ T cells in the blood were also detected and are shown in Figure S3a in the Supporting Information, which presents the similar variation of CD3^+^ T cells in the spleen. The ratio of CD3^+^ T cells in the BM and blood was also calculated and is shown in Figure S3b in the Supporting Information. A high ratio (up to 6) for GBM‐bearing mice clearly demonstrates the sequestration of T cells in the BM. The similar ratios of CD3^+^ T cells in the BM and blood (around 2) observed in the healthy mice and the GBM‐bearing mice treated with PX prove the capability of PX releasing the sequestrated T cells in the BM. The CD3^+^ T cells in the lymph nodes were also characterized by flow cytometry analysis (Figure S4a, Supporting Information) and immunofluorescence images (Figure S4b, Supporting Information), of which T cells in the lymph nodes of GBM‐bearing mice were much lower than those in the healthy mice, and were increased to the normal level after they were treated with PX. These results further demonstrate the fact that PX can improve the expression of S1PR1 on T cells to afford them egressing from the BM to the lymph node.

**Figure 2:**
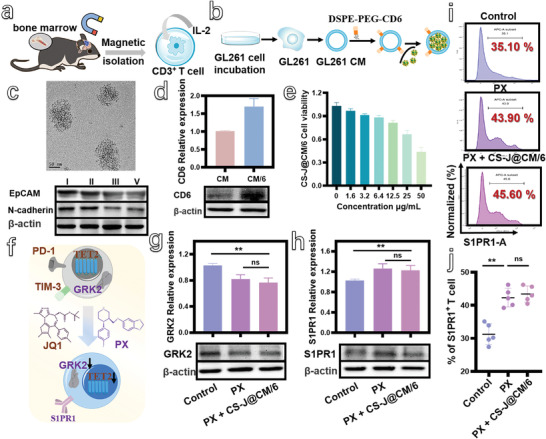
a) Schematic illustration of naïve CD3^+^ T cells isolated from GBM‐bearing C57BL/6 mice. b) Schematic illustration of preparation of CS‐J@CM/6 NPs. c) TEM image of CS‐J@CM/6 NPs and Western blot analysis of specific proteins on the membrane, including EpCAM and N‐cadherin from I) GL261 cell lysate, II) GL261 cell membrane vesicles, III) CM/6 cell membrane vesicles, and V) CS‐J@CM/6 NPs. d) Detection of CD6 incorporated into cell membrane. e) Viability of CD3^+^ T cells cultured with different concentrations of CS‐J@CM/6 NPs. f) Schematic illustration of the influence of PX and JQ1 on the receptors of CD3^+^ T cells. g,h) Detection of GRK2 expression and S1PR1 expression by T cells. i,j) Flow cytometry analysis of S1PR1^+^T cells and their percentage after CD3^+^ T cells were cultured with PX alone, or with PX and CS‐J@CM/6 NPs.

To investigate the role of PX in stabilizing S1PR1 on the T cell surface to free the sequestered T cells in the BM, and the role of the biomimetic nanoparticles in activating the antitumor immune response and preventing T cell exhaustion, we obtained naïve CD3^+^ T cells from the BM and prepared the biomimetic nanoparticles for the following experiments (**Figure** **2**a). The ultrasmall Cu_2−_ *_x_* Se nanoparticles were synthesized in an aqueous solution at ambient conditions, and then modified with *β*‐cyclodextrin (CD) (named as Cu_2−_ *_x_* Se‐CD nanoparticles, CS NPs) for loading the hydrophobic drug JQ1 (referred to CS‐JQ1 nanoparticles, CS‐J NPs).^[^ ^39^, ^40^ ^]^ To improve the nanoparticle ability to cross the BBB and target tumor cells, we used the GL261 cell membranes (CMs) conjugated with CD6 to coat the CS‐J NPs to result in CS‐J@CM/6 NPs (Figure 2b and Figure S5a, Supporting Information). The modified membrane can localize together with the ALCAM expressed by the endothelial cells of BBB and then target tumor cells through the homologous adhesion effect.^[^ ^41^ ^]^ As shown by the transmission electron microscope (TEM) images in Figure S5b in the Supporting Information and Figure 2c, the monodispersed CS‐J NPs had a very small size (3.2 ± 0.6 nm). They were assembled into large clusters (64 ± 3.5 nm) after being coated with CMs. Their high‐resolution TEM image is inserted in Figure S5b in the Supporting Information, which clearly displays lattice fringes with an interplanar spacing of 0.20 nm, matching with that of the (220) planes of cubic berzelianite (Cu_2−_ *_x_* Se). Furthermore, as shown in Figure S5c in the Supporting Information, their crystal structure was determined by powder X‐ray diffraction, which showed the characteristic diffraction peaks of cubic berzelianite (Cu_2−_ *_x_* Se, JCPDS no. 06–0680). The broad diffraction peaks are attributed to their ultrasmall size. Their hydrodynamic size was determined by the dynamic light scattering method and is shown in Figure S5d in the Supporting Information. Obviously, after being coated with CM/6, the hydrodynamic size of nanoparticles was increased from 10 to 100 nm. Their polydispersity index was 0.141 and 0.187, respectively. In addition, the zeta potential of CS NPs in Figure S5e in the Supporting Information was −9.8 mV, and reached to −6.5 mV after modification with JQ1 (i.e., CS‐J NPs). After being coated with CM/6, the zeta potential of nanoparticles (i.e., CS‐J@CM/6 NPs) was decreased to −15.8 mV. The variations of particle size and zeta potential prove that JQ1 was successfully loaded onto CS NPs and CM/6 was also coated onto the nanoparticles.

To further show the successful coating of cancer cell membrane onto the nanoparticles, we used Western blotting to characterize the cell adhesion molecules (EpCAM and N‐cadherin) on cancer cell membrane. As shown in Figure 2c, the GL261 cell lysate, GL261 cell membrane vesicles, CM/6 cell membrane vesicles, and CS‐J@CM/6 NPs possessed the similar EpCAM and N‐cadherin profiles on the cell membrane for adhesion. These results illustrate the good retention of proteins and their lower damage during coating process, and demonstrate that the CD6 was successfully incorporated into GL261 CM through the aid of lipid tethers (Figure 2d).

To explore the influence of surface functionalization and coating on T cells, we used the methyl thiazolyl tetrazolium (MTT) assay to investigate the cytotoxicity of resultant CS‐J@CM/6 NPs toward CD3^+^ T cells. As shown in Figure 2e, the cytotoxicity of CS‐J@CM/6 NPs was relatively lower when the copper concentration was below 12.5 µg mL^−1^. Furthermore, the cytotoxicity of JQ1 and PX to CD3^+^ T cells is presented in Figure S6a,b in the Supporting Information for comparison, in which both JQ1 and PX exhibited a low toxicity to CD3^+^ T cells when their concentrations were smaller than 5 and 2.5 µg mL^−1^, respectively. In addition, BV2 cells (microglia of mouse) and SH‐SY5Y cells (neuroblastoma of mouse) were used to assess the toxicity of PX to non‐T cells in the brain (Figure S6c,d, Supporting Information). After BV2 cells or SH‐SY5Y cells were cultured with PX for 12 h, their viability was higher than that of GL261 cells under the same PX concentration. These results illustrate that PX is less toxic to non‐T cells than to tumor cells in the brain.

Within the safe range of PX and CS‐J@CM/6 NPs, the mechanisms of PX stabilizing S1PR1 on the T cell surface and the biomimetic CS‐J@CM/6 NPs preventing T cell exhaustion were studied (Figure 2f). The expression of GRK2 in the BM‐derived CD3^+^ T cells is shown in Figure 2g. Obviously, the GRK2 expression was greatly decreased after the cells were incubated with PX alone or PX with CS‐J@CM/6 NPs for 12 h. The result illustrates that the GRK2 was successfully inhibited by PX, because it can serve as a selective serotonin reuptake inhibitor (SSRI), and bond with the active site of GRK2 to form PX‐G*βγ* complex.^[^ ^33^, ^42^ ^]^ The expression of S1PR1 by CD3^+^ T cells was also detected and is shown in Figure 2h. Obviously, PX improved the S1PR1 expression to almost 1.2‐fold of that from the Control group. In addition, the CS‐J@CM/6 NPs had no influence on the expression of S1PR1 on T cells. The S1PR1 expression was also characterized by the flow cytometry. Clearly, after CD3^+^ T cells were cultured with PX or PX + CS‐J@CM/6 NPs (Figure 2i,j), the S1PR1^+^CD3^+^ T cells were increased from 35.10% to 43.90% and 45.60%, respectively, which suggests that PX improved the S1PR1 expression on T cells through inhibiting the GRK2 expression. This is consistent with the Western blot results in Figure 2h.

The above results confirm that the antidepressant PX as an SSRI can decrease the GRK2 expression to stabilize the surface S1PR1 on T cells for freeing them from the BM of GBM‐bearing mice for immunotherapy (Figure 2f). To activate T cells, it is necessary to release tumor‐associated antigens through ICD of tumor cells. It has been proved that the Cu_2−_ *_x_* Se NPs can react with H_2_O_2_ to generate ROS to induce the ICD of tumor cells for releasing the damage‐associated molecular patterns (DAMPs), which activated antigen‐presenting cells (APCs), especially dendritic cells (DCs) to stimulate tumor‐specific effector T cells.^[^ ^43^ ^]^ Therefore, we evaluated the Fenton‐like property of CS‐J@CM/6 NPs and used a fluorescent probe to detect the generation of ROS. Compared with the case of pure H_2_O_2_, very strong fluorescence at 529 nm was observed when it reacted with the CS‐J@CM/6 NPs (Figure S7a, Supporting Information), which supports that CS‐J@CM/6 NPs can degrade H_2_O_2_ (Figure S7b, Supporting Information) to generate ROS. Furthermore, there was about 5.6 ppm O_2_ generated from the degradation of H_2_O_2_ catalyzed by CS‐J@CM/6 NPs (Figure S7c, Supporting Information), which was more than that (0.5 ppm O_2_) produced without CS‐J@CM/6 NPs.

The above results highlight the potential of CS‐J@CM/6 NPs in degrading endogenous H_2_O_2_ within tumor to generate ROS and O_2_ through the Fenton‐like reaction.^[^ ^37^, ^39^, ^40^ ^]^ Their capability of degrading H_2_O_2_ in the tumor cell environment was further assessed with G261 cells. Similar to the CD3^+^ T cells, the viability of G261 cells cultured with CS‐J or CS‐J@CM/6 NPs was investigated by the MTT assay. As shown in Figure S8a in the Supporting Information, the cytotoxicity of CS‐J NPs was relatively low when the concentration was below 12.5 µg mL^−1^. In contrast, CS‐J@CM/6 NPs showed high toxicity when their concentration was 12.5 µg mL^−1^ (Figure S8b, Supporting Information) because more nanoparticles were uptaken due to the homologous adhesion effect of tumor cell membrane. The uptake of CS‐J@CM/6 NPs by tumor cells was determined by the inductively coupled plasma‐mass spectrometry (ICP‐MS). Figure S8c in the Supporting Information shows 20% increment of Cu in the tumor cells cultured with CS‐J@CM/6 NPs, as compared with that of cells cultured with CS‐J NPs. The results further prove that CS‐J@CM/6 NPs can effectively target the tumor cells to induce their apoptosis. In addition, the toxicity of PX to GL261 cells in Figure S9a in the Supporting Information illustrates that PX had less toxicity to GL261 cells with a concentration smaller than 5 µg mL^−1^. When the same concentration of PX (5 µg mL^−1^) was jointly used with CS‐J@CM/6 NPs, the toxicity was slightly higher than that of CS‐J@CM/6 NPs alone, illustrating that PX (5 µg mL^−1^) had a minor influence on the GL261 cells (Figure S9b, Supporting Information).

**Figure 3:**
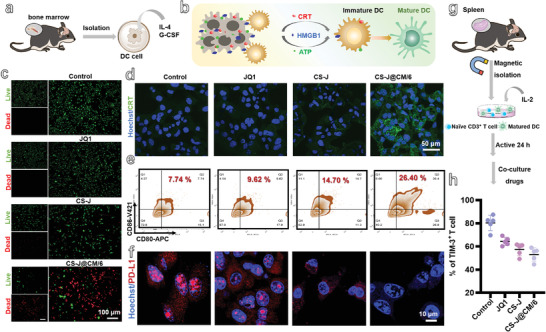
a) Schematic illustration of DCs isolated from healthy C57BL/6 mice. b) Schematic illustration of the DAMPs (CRT, ATP, and HMGB1) released from the dying tumor cells to promote the DC maturation, triggered by large amount of ROS generated from the degradation of H_2_O_2_ by CS‐J@CM/6 NPs. c) Fluorescence images of GL261 cells stained with a live/dead kit after different treatments (scale bar: 100 µm). d) Immunofluorescence images of CRT exposure on GL261 cells (scale bar: 50 µm). e) Flow cytometry analysis of matured DCs. f) Fluorescence images of PD‐L1 on GL261 cells (scale bar: 10 µm). g) Schematic illustration of CD3^+^ T cells isolated from C57BL/6 mice and stimulated with matured DCs. h) The percentage of TIM‐3^+^ T cells.

To determine the apoptotic tumor cells induced by ROS generated from the Fenton‐like reaction of nanoparticles, the apoptosis rates of GL261 cells cultured with JQ1, CS‐J NPs, and CS‐J@CM/6 NPs were quantified to be 34.97%, 45.73%, and 76.82% by flow cytometry, respectively (Figure S10, Supporting Information). Obviously, after cultured with CS‐J@CM/6 NPs, the corresponding apoptotic cells were increased by 1.7‐fold in comparison with those cultured with CS‐J NPs. Furthermore, live/dead staining of GL261 cells also demonstrates that the cells were killed by ROS generated by the Fenton‐like reaction of CS‐J@CM/6 NPs (**Figure** **3**c), which is consistent with the results shown in Figure S10 in the Supporting Information. All these results illustrate that the CS‐J@CM/6 NPs can generate ROS to induce GL261 cells apoptosis.

Based on these results, we detected the release of DAMPs to induce the maturation of DCs isolated from the BM of C57BL/6 mice (Figure 3a,b). The calreticulin (CRT) exposure and HGBM1 released from GL261 cells were detected by immunofluorescence, after they were cultured with CS‐J@CM/6 NPs. As shown in Figure 3d, there was no obvious CRT on the membrane of GL261 cells after they were cultured with JQ1 or CS‐J NPs, demonstrated by the very weak green fluorescence. However, after they were incubated with CS‐J@CM/6 NPs, the GL261 cells showed stronger green fluorescence, which proves the ICD and exposure of CRT caused by the CS‐J@CM/6 NPs. Furthermore, the HGBM1 released from the tumor cells cultured with CS‐J@CM/6 NPs was higher than that of cells cultured with JQ1, CS‐J, or CS‐J@CM/6 NPs (Figure S11, Supporting Information). The results quantified from Western blot and flow cytometry (Figure S12, Supporting Information) are consistent with these in Figure 3d and Figure S11 in the Supporting Information. All the results illustrate that CS‐J@CM/6 NPs can trigger the ICD of GL261 cells and stimulate CRT exposure and HGBM1 release through ROS generated by the Fenton‐like reaction.

The maturated DCs induced by these DAMPs are evaluated in Figure 3e. After BM‐derived DCs (bone marrow dendritic cells, BMDCs) were co‐incubated with GL261 cells, which were pretreated with CS‐J@CM/6 NPs, they showed the strongest immunogenicity and induced the highest matured DCs (CD11c^+^CD80^+^CD86^+^, 26.40%), compared to those cultured with CS‐J NPs (14.70%), and those from the Control group (7.74%). The results illustrate that the maturation of DCs was significantly enhanced by the ICD induced by CS‐J@CM/6 NPs because of the homologous adhesion effect, which is significant for tumor immunotherapy.^[^ ^44^ ^]^

As persistent antigen stimulation can exhaust T cells and the terminally exhausted T cells (PD‐1^+^TIM‐3^+^ T) lose the capability of killing tumor cells. Therefore, we investigated whether the CS‐J@CM/6 NPs could prevent the T cell exhaustion. As the PD‐L1 expressed by tumor cells can restrain the activity of T cells at tumor site, preventing T cell exhaustion is crucial after the stimulation of tumor‐specific effector T cells. We characterized the expression of PD‐L1 on GL261 cells by immunofluorescence (Figure 3f), Western blot (Figure S13a, Supporting Information), and flow cytometry (Figure S13b, Supporting Information). All results prove that the CS‐J@CM/6 NPs decreased PD‐L1 expression on GL261 cells.

Apart from tumor cells can decrease the activity of T cells, the T cells themselves can be exhausted under persistent antigen simulation, due to their increased expressions of PD‐1 and TIM‐3. Therefore, it is also very important to decrease the PD‐1 and TIM‐3 expression on T cells for preventing the T cell exhaustion and improving their activity.^[^ ^45^ ^]^ It has been demonstrated that JQ1 as an extra‐terminal BET family inhibitor can smartly decrease the TET2 expression to simultaneously decrease the PD‐1 and TIM‐3 expression, which are markers of exhausted T cells.^[^ ^31^ ^]^ As shown in Figure 3g, naïve CD3^+^ T splenocytes were magnetically isolated from the spleen of C57BL/6 mice, followed by stimulation with matured DCs treated with CS‐J@CM/6 NPs. After 24 h activation, CD3^+^ T cells were incubated with the CS‐J@CM/6 NPs to determine their TIM‐3 and PD‐1 expression by flow cytometry (Figure 3h and Figure S14, Supporting Information).^[^ ^46^ ^]^ The results demonstrate that the PD‐1 expression on T cells was decreased from 73.90% to 40% after cultured with CS‐J@CM/6 NPs. Furthermore, the TIM‐3 expression (Figure 3h) also proves that the CS‐J@CM/6 NPs decreased more than 23% of TIM‐3 expression on T cells. These results indicate that the CS‐J@CM/6 NPs can not only decrease the PD‐L1 expression on CL261 cells, but also decrease the PD‐1/TIM‐3 expression on T cells, which prevented the T cell exhaustion caused by tumor immunosuppressive microenvironment and persistent antigen simulation (Figure 2f).

The above in vitro results demonstrate that the CS‐J@CM/6 NPs can not only stimulate tumor‐specific effector T cells through the ROS generated from their Fenton‐like reaction, but also can prevent T cell exhaustion caused by the persistent antigen stimulation. To demonstrate the capability of CS‐J@CM/6 NPs crossing the BBB, we established a transwell BBB model by seeding the bEnd.3 cells in the upper chamber and the GL261 cells on the lower chamber (Figure S15a, Supporting Information). When the transepithelial electrical resistance (TEER) values reached to 150 Ω cm^2^, the CS‐J NPs, CS‐J@CM NPs, or CS‐J@CM/6 NPs were then added in the upper chambers. After 4 h incubation, the TEER value was analyzed (Figure S15b, Supporting Information). Obviously, there was no difference in TEER results, illustrating the integrity of mimic BBB composed by bEnd.3 cells. In addition, the GL261 cells in the lower chambers were collected to determine the Cu concentration by ICP‐MS (Figure S15c, Supporting Information). Obviously, the Cu concentration in the tumor cells from the CS‐J@CM/6 group was about 1.5‐fold of that in the Control group. In addition, the other groups of cells showed no increase in Cu concentration compared with the Control group. The results indicate that CS‐J@CM/6 NPs can cross the BBB and accumulate in the GL261 cells through the specific interactions between CD6 and ALCAM in vitro. Furthermore, the in vivo remarkable red fluorescence of ALCAM in the tumor tissue is presented in Figure S15d in the Supporting Information, which illustrates the high expression of ALCAM. The high expression of ALCAM in the brain was further demonstrated by the Western blot results (Figure S15e, Supporting Information).

**Figure 4:**
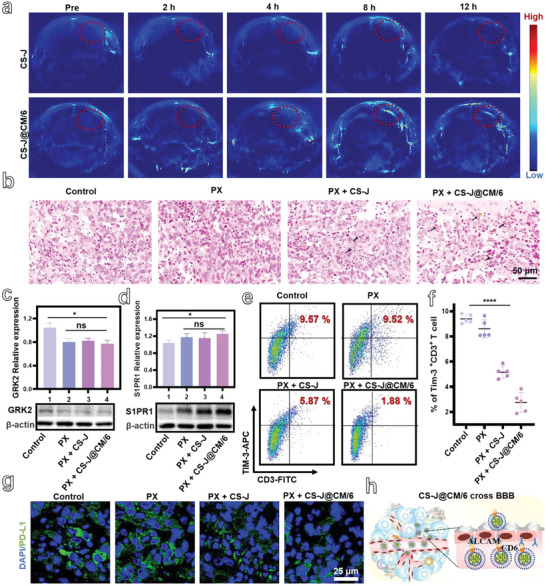
a) Photoacoustic (PA) imaging of tumors from orthotopic GL261 tumor‐bearing mice collected before and after tail vein injection of CS‐J NPs or CS‐J@CM/6 NPs (dose: 5 mg kg^−1^) at different time points (tumors are highlighted by the red dashed circles). b) Imaging of tumor slices stained with rubeanic acid (RA) from GL261 tumor‐bearing mice administered with CS‐J NPs or CS‐J@CM/6 NPs (scale bar: 50 µm). c,d) Detection of GRK2 expression and S1PR1 expression on the CD3^+^ T cells in the BM of mice from different groups (*n* = 3). e,f) Flow cytometry analysis of TIM‐3^+^CD8^+^ T cells and their percentage in tumor from different group of mice (*n* = 5). g) Immunofluorescence images of PD‐L1 expression in tumor slices (scale bar: 25 µm). h) Schematic illustration of the specific interaction between ALCAM expressed on the endothelial cells of BBB and CD6 on the CS‐J@CM/6 NPs to make nanoparticles cross the BBB and target tumor.

Because Cu_2−_ *_x_* Se NPs can efficiently convert the near‐infrared (NIR) light into heat for photoacoustic (PA) imaging, the PA imaging was used to characterize the accumulation of CS‐J@CM/6 NPs at tumor site. The PA images of brain were collected at different time points after the GL261 tumor‐bearing mice were intravenously injected with CS‐J NPs or CS‐J@CM/6 NPs (**Figure** **4**a). The PA signal of tumor in the mice injected with CS‐J@CM/6 NPs reached to the maximum at 8 h postinjection, which was higher than that of mice administrated with CS‐J NPs unmodified with cell membrane and CD6. The results prove that the CD6 endowed CS‐J@CM/6 NPs with excellent capability of crossing the BBB through its strong affinity to ALCAM expressed on the BBB to enhance nanoparticle accumulation through the homologous adhesion of tumor cell membrane (Figure 4h). Furthermore, the staining of tumor slices clearly showed that copper stains in the tumor slices of mice injected with CS‐J@CM/6 NPs were much more than those of mice injected with CS‐J NPs (Figure 4b).^[^ ^29^ ^]^ The result is consistent with the PA images in Figure 4a, illustrating that the CS‐J@CM/6 NPs can cross the BBB to accumulate at tumor site. To detect the biodistribution of CS‐J@CM/6 NPs after intravenous administration into GBM‐bearing mice, we dissected the mice at 1, 3, 5, 7, and 15 days postinjection to harvest their major organs for measuring the copper concentrations by ICP‐MS. As shown in Figure S16a in Supporting Information, the CS‐J@CM/6 NPs were mainly distributed in the reticuloendothelial organs such as liver, spleen, and lung. In addition, the high copper concentration in the kidney suggests that some CS‐ J@CM/6 NPs were excreted through renal clearance.

To determine whether the PX was able to increase the accumulation of nanoparticles in the brain tumor, we detected the copper concentration in the tumor from different groups of mice by ICP‐MS (Figure S16b, Supporting Information). Obviously, the copper concentration from the CS‐J@CM/6 group was similar to that from the PX + CS‐J@CM/6 group, which was about 1.89‐fold of that from the Control group. In addition, we also evaluated the in vivo release of T cells sequestrated in the BM of GBM‐bearing mice through PX. First, GRK2 expression on T cells in the BM of tumor‐bearing mice from the Control group, PX group, PX + CS‐J group, and PX + CS‐J@CM/6 group was detected by the Western blot. As displayed in Figure 4c, the GRK2 expression in the GBM‐bearing mice orally administrated with PX (10 mg kg^−1^, every 2 days) was obviously decreased more than 20% as compared with the tumor‐bearing mice treated without PX. In addition, the NPs had no influence on the inhibition of GRK2 expression. Second, the S1PR1 expression was also detected by Western blot and is shown in Figure 4d, the S1PR1 expression of tumor‐bearing mice after treatment with PX was greatly increased to almost 1.2‐fold. In addition, the ratio of S1PR1^+^CD3^+^ T cells was increased from 3.89% to 5.53% (Figure S17, Supporting Information), which is consistent with the results in Figure 4d.

The above all results prove that PX can stabilize the S1PR1 on T cells by inhibiting GRK2 expression in the BM.^[^ ^47^ ^]^ Apart from the reversing sequestration of T cells in the BM, the influence of different treatments on the T cell exhaustion in GBM‐bearing mice was assessed by measuring the expression of TIM‐3. As shown in Figure 4e,f, the TIM‐3^+^CD3^+^ T cells in tumor after different treatments were detected by flow cytometry. Obviously, the JQ1 loaded on CS‐J NPs decreased the ratio of TIM‐3^+^CD3^+^ T cells from 9.57% to 5.87%. TIM‐3^+^CD3^+^ T cells in the GBM‐bearing mice treated with CS‐J@CM/6 NPs was 1.88%, which was decreased by almost 75% in comparison with that of tumor‐bearing mice injected with CS‐J NPs because of the capability of CS‐J@CM/6 NPs crossing the BBB and targeting tumor.

Apart from TIM‐3, PD‐1 is also an important receptor for exhausted T cells.^[^ ^48^, ^49^, ^50^ ^]^ Figure S18a in the Supporting Information shows that the PD‐1^+^CD3^+^ T cells were decreased from 26.4% to 13.9% after the GBM‐bearing mice were treated with PX and CS‐J@CM/6 NPs. The effects of CS‐J@CM/6 NPs on tumor cells were also detected through immunofluorescence analysis. As shown in Figure 4g, the fluorescence of PD‐L1 in the tumor slices of mice treated with PX + CS‐J@CM/6 NPs was much weaker than that of tumor‐bearing mice received other treatments, which indicates the decreasing PD‐L1 expression in tumor slices of mice administrated with both PX and CS‐J@CM/6 NPs, because of the release of JQ1 from the accumulated CS‐J@CM/6 NPs at tumor site. Furthermore, the PD‐L1 expression was also detected by the flow cytometry (Figure S18b, Supporting Information), which clearly showed the obvious decrease of PD‐L1 in the GBM‐bearing mice treated with PX + CS‐J@CM/6 NPs as compared with tumor‐bearing mice received other treatments. These results prove that the CS‐J@CM NPs can prevent T cell exhaustion effectively through simultaneously decreasing PD‐1 and TIM‐3 expression on T cells and PD‐L1 expression on tumor cells.

**Figure 5:**
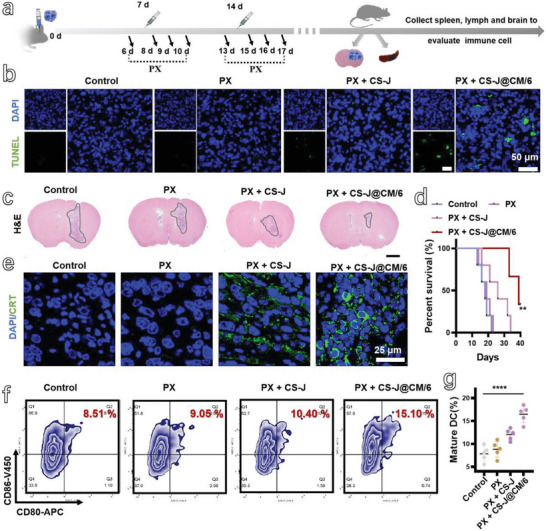
a) Schematic illustration of the immunotherapy of orthotopic GL261 tumor by using PX and CS‐J NPs or CS‐J@CM/6 NPs. b) The TUNEL (green fluorescence) for characterizing the apoptotic cells, in which the nuclei were stained with 4′,6‐diamidino‐2‐phenylindole (DAPI, blue fluorescence) (scale bar: 50 µm). c) H&E staining images of brain slices from different groups of mice after being treated for 20 days (scale bar: 1 mm). d) Survival rates of mice from different groups (*n* = 5). e) Immunofluorescence images of CRT exposed in tumor (scale bar: 25 µm). f,g) Flow cytometry analysis of matured DCs (CD11c^+^CD80^+^CD86^+^) and their percentage in the CLNs of mice from different groups (*n* = 5).

Based on the above results, we evaluated the antitumor effects of different treatments on four groups of mice bearing orthotopic GBM (i.e., Control group, PX group, PX + CS‐J group, and PX + CS‐J@CM/6 group). As shown in **Figure** **5**a, the Control group of GBM‐bearing mice did not receive any treatment. The PX group of GBM‐bearing mice were orally administrated with PX (dose: 10 mg kg^−1^) from day 6 to day 10, and then followed by another cycle from day 13 (Figure 5a). The GBM‐bearing mice in the groups of PX + CS‐J and PX + CS‐J@CM/6 were received PX treatment as above, and then injected with CS‐J NPs solution or CS‐J@CM/6 NP solution (dose: 5 mg kg^−1^) at day 7 and day 14. After different treatments, the stronger green fluorescence in terminal deoxynucleotidyl transferase dUTP nick‐end labeling (TUNEL) analysis (Figure 5b) indicates that the tumor cell apoptosis was significantly increased in the tumor‐bearing mice treated by joint use of PX and CS‐J@CM/6 NPs.^[^ ^51^ ^]^ In addition, there was almost no green fluorescence in the tumor slices of mice from the Control group, PX group, and even the PX + CS‐J NPs group, which illustrates that the CS‐J@CM/6 NPs can efficiently accumulate at tumor site to damage the cancer cells.

To further demonstrate the antitumor performance of CS‐J@CM/6 NPs combined with PX, the hematoxylin and eosin (H&E) staining of brain slices from four different groups of mice after 3 days treatment illustrates that the tumor from the PX + CS‐J@CM/6 group of mice was the smallest among the four groups of mice (Figure 5c). All the results further prove that jointly using “old drug” PX and biomimetic CS‐J@CM/6 NPs can induce cancer cells apoptosis for oncotherapy. In addition, the survival rates of mice were also recorded in Figure 5d, in which the survival rate of the mice from PX + CS‐J@CM/6 group was much higher than that of other groups of mice, evidenced by 60% of mice lived more than 40 days after implantation of GL261 cells. The results further prove that the CS‐J@CM/6 NPs combined with PX can prolong the lifetime of GBM‐bearing mice because of the better therapeutic efficacy. The body weights were also recorded every other day (Figure S19, Supporting Information), and the decrease of mouse body weights with the tumor progression in the group of PX + CS‐J@CM/6 was much slower than that of mice from other groups. Furthermore, the bioluminescence in the mouse brain was also detected at day 7, 10, 20, and 30 after treatment (Figure S20, Supporting Information), the bioluminescence of brain in the mice from the PX + CS‐J@CM/6 group was the weakest in four groups. These results demonstrate that the CS‐J@CM/6 NPs can cross BBB for GBM immunotherapy and achieve excellent therapy performance when combined with PX.

It is also very important to explore whether the excellent therapeutic efficacy was achieved by the antitumor immunity. The ICD of tumor cells for stimulating tumor‐specific effector T cells was detected and is shown in Figure 5e. Obviously, the green fluorescence of CRT in tumor slices from the PX + CS‐J@CM/6 group was rather stronger than that of other groups, which illustrates that the CS‐J@CM/6 NPs can cross the BBB to accumulate at tumor site and induce the ICD of tumor cells to expose the CRT and activate antitumor immunity. Furthermore, the HGBM1 in Figure S21 in the Supporting Information demonstrates that the combination of PX and CS‐J@CM/6 NPs caused the most effective ICD in tumor, proved by the strongest green fluorescence of the released HGBM1, which is consistent with the CRT result in Figure 5e. Furthermore, the matured DCs (CD11c^+^CD86^+^CD80^+^) (Figure S22a, Supporting Information) in the CLNs from four groups of mice are shown in Figure 5f,g, in which about 15.10% of matured DCs were found in the lymph nodes of mice from PX + CS‐J@CM/6 group, and they were 1.7‐ and 1.5‐fold higher than those from the Control group and PX + CS‐J group, respectively. The ratios of matured DCs in tumor from different groups of mice were also detected (Figure S22b, Supporting Information). Obviously, the ratio of matured DCs was increased from 3.32% for the Control group of mice to 29.90% for mice from the PX + CS‐J@CM/6 group, which further illustrates that the CS‐J@CM/6 NPs can induce ICD of tumor cells to expose CRT and release HGBM1 to activate maturation of DCs.

**Figure 6:**
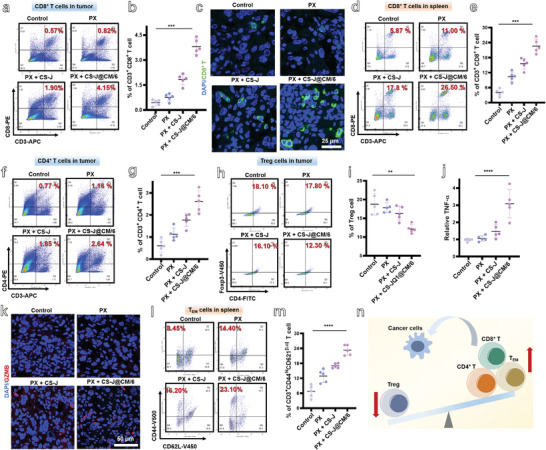
a) Flow cytometry analysis of CD8^+^ T cells (CD3^+^CD8^+^) in tumor from different groups of mice (Control group; PX group; PX + CS‐J group; PX + CS‐J@CM/6 group). b) The percentage of CD8^+^ T cells in tumor (*n* = 5). c) Immunofluorescence images of CD8^+^ T cells in tumor (scale bar: 25 µm). d,e) Flow cytometry analysis of CD8^+^ T cells (CD3^+^CD8^+^) and their percentage in the spleen of mice from different groups (*n* = 5). f,g) Flow cytometry analysis of CD4^+^ T cells (CD3^+^CD4^+^) and their percentage in the tumor of mice from different groups (*n* = 5). h,i) Flow cytometry analysis of Treg cells (CD3^+^CD25^+^CD4^+^Foxp3^+^) and their percentage in tumor of mice from different groups (*n* = 5). j) Tumor necrosis factor alpha (TNF‐*α*) secreted in the serum analyzed by ELISA (*n* = 5). k) Immunofluorescence images of GZMB^+^ (GranzymeB^+^) T cells in tumor (scale bar: 50 µm). l,m) Flow cytometry analysis of T_EM_ cells (CD3^+^CD44^hi^CD62L^low^) and their percentage in the spleen of mice from different groups after implantation of tumor cells for 20 days (*n* = 5). n) Schematic illustration of the change of immunity cells after treatment.

As the matured DCs can active T cells for immunotherapy, the immunity effect was also characterized by the infiltration of CD8^+^ T cells in the tumor, spleen, and lymph nodes after 20 days treatment. The immunocompetent GL261 tumor mice were also similarly divided into four groups, i.e., Control, PX, PX + CS‐J, and PX + CS‐J@CM/6 groups. As shown in **Figure** **6**a,b, 1.90% CD8^+^ T cells had been activated and infiltrated into the tumor in the PX + CS‐J group of mice, which were almost 3.3‐fold higher than that of tumor‐bearing mice from the Control group. The CD8^+^ T cells in the tumor from the PX + CS‐J@CM/6 group of mice were almost 2.2‐fold higher than those from the PX + CS‐J group, further demonstrating that CS‐J@CM/6 NPs can cross the BBB and effectively elicit the infiltration of CD8^+^ T cells into tumor after joint use with PX. The remarkable green fluorescence of CD8^+^ T cells in the tumor slices from the PX + CS‐J@CM/6 group of mice (Figure 6c), in comparison with the absence of green fluorescence from the other groups, further proves the enhancement of the activated and infiltrated intratumoral CD8^+^ T cells achieved by combination of PX and CS‐J@CM/6 NPs. Apart from the tumor site, the change of CD8^+^ T cells in the spleen of four groups of mice (Figure 6d,e) further supports the activation of antitumor immunity. Obviously, the CD8^+^ T cells in the spleen were increased from 5.87% for the Control group of mice to 11.00% for the PX group of mice. The drastic increase of CD8^+^ T cells in the spleen is attributed to the fact that PX can release the sequestrated T cells from BM through stabilizing their surface S1PR1 receptor. In addition, the ratio of CD8^+^ T cells in the spleen of tumor‐bearing mice from the PX + CS‐J@CM/6 group was increased to 26.5%, which was almost 2.4‐ and 1.5‐fold of that from the PX group and PX + CS‐J@CM/6 group. The results demonstrate that joint use of PX and CS‐J@CM/6 NPs can effectively stimulate tumor‐specific effector T cells in the GBM‐bearing mice.

In addition to CD8^+^ T cells, the CD4^+^ T cells also play an important role in immunotherapy. The CD4^+^ T cells in tumor from four groups of mice were detected and are shown in Figure 6f,g, where about 2.64% of CD4^+^ T cells were in the tumor from the PX + CS‐J@CM/6 group of mice, which was 3.4‐ and 1.4‐fold higher than those from the Control group and PX + CS‐J group, respectively. The ratio of CD4^+^ T cells in the spleen of four groups of GBM‐bearing mice was also detected (Figure S23, Supporting Information). There were about 24.9% of CD4^+^ T cells in the spleen of mice from the PX + CS‐J@CM/6 group, which was about 1.7‐ and 1.2‐fold of that from the Control group and PX + CS‐J group. All the results illustrate that PX combined with CS‐J@CM/6 NPs increased both the CD8^+^T cells and CD4^+^ T cells to enhance the immunotherapy efficiency.

The immunosuppressive Treg cells (CD3^+^CD4^+^CD25^+^Foxp3^+^cells) (Figure S24a, Supporting Information) in tumor from the four groups of mice were also detected. There were 18.1% and 17.8% of Treg cells in tumor from the Control group and PX group (Figure 6h,i). However, the Treg cells in the tumor from the PX + CS‐J and PX + CS‐J@CM/6 groups were decreased to 16.1% and 12.3%, respectively. Moreover, the change of Treg cells at lymph nodes was also detected. As shown in Figure S24b,c in the Supporting Information, less than 0.54% Treg cells were observed in the PX + CS‐J@CM/6 group, which were almost one‐third of those obtained from Control group. These results illustrate that combination of PX and CS‐J@CM/6 NPs can not only induce the infiltration of CD8^+^ T cells in tumor, but also decrease the immunosuppressive Treg cells which suppressed the therapeutic effect for GBM.

In addition to T cells, the cytokines of tumor necrosis factor alpha (TNF‐*α*) in the serum secreted by the activated immune cells were analyzed through enzyme‐linked immunosorbent assay (ELISA, Figure 6j). The TNF‐*α* from the serum of mice in the PX + CS‐J@CM/6 group was about 3‐ and 1.5‐fold higher than that from the Control group and the PX + CS‐J group, respectively, which again proves that joint use of PX and CS‐J@CM/6 NPs can efficiently induce the infiltration and activation of immune cells. Furthermore, we investigated the effects of CS‐J@CM/6 NPs on granzyme B (GZMB) expression at tumor site, which is a toxic molecule released by active T cells.^[^ ^52^, ^53^, ^54^, ^55^ ^]^ As shown in Figure 6k, the staining immunofluorescence of GZMB in the tumor slices from mice in the CS‐J@CM/6 NPs group was stronger than that of Control group, which illustrates that CS‐J@CM/6 NPs can improve the activity of T cells at tumor site through the JQ1.

To investigate whether the joint use of PX and CS‐J@CM/6 NPs could activate the memory immunity, we detected the memory T cells (T_EM_, CD3^+^CD44^hi^CD62L^low^) in the spleen of mice by flow cytometry (Figure S25, Supporting Information and Figure 6l,m). The T_EM_ cells in the spleen from the Control group of mice were 8.45%, which was obviously increased up to 23.10% in the spleen of mice from the PX + CS‐J@CM/6 group. The notable increment in the T_EM_ cells from the PX + CS‐J@CM/6 group (2.7‐fold of that from the Control group) demonstrates that joint use of PX and the CS‐J@CM/6 NPs improved immunotherapeutic effect with the induced memory T cells. Overall, the joint use of PX and CS‐J@CM/6 NPs can improve the infiltration of antitumor immune cells (CD8^+^ T, CD4^+^ T and T_EM_) in tumor and decrease the immunosuppressive Treg cells to improve the immunotherapy efficacy, as schematically represented in Figure 6n.

In addition to the excellent antitumor efficacy, the in vivo biocompatibility of CS‐J@CM/6 NPs was assessed by H&E staining of the major organs (i.e., heart, liver, spleen, lung, kidney) of mice, which were sacrificed at 18 days posttreatment. There was no obvious damage to the major organs after injection of CS‐J@CM/6 NPs, in comparison with healthy mice (Figure S26, Supporting Information), which demonstrates the good biocompatibility of CS‐J@CM/6 NPs.

## 3 Conclusion

In summary, we demonstrate the significance of increasing T cell infiltration and reversing their dysfunction in tumor to improve the GBM immunotherapy by jointly using “old drug” PX and multifunctional biomimetic CS‐J@CM/6 nanoparticles. The PX can release T cells sequestrated in the BM by stabilizing their surface S1PR1 receptor through the inhibition of GRK2, and increasing their infiltration in tumor. The biomimetic CS‐J@CM/6 nanoparticles can effectively cross the BBB through the strong affinity of surface CD6 to ALCAM expressed by microvascular endothelium of BBB. The loaded JQ1 can decrease the expression of PD‐1 and TIM‐3 on T cells and PD‐L1 on tumor cells to ameliorate T cell exhaustion and dysfunction. The synergetic roles of PX and biomimetic CS‐J@CM/6 nanoparticles drastically improved infiltration of antitumor immune cells (CD8^+^ T, CD4^+^ T, and T_EM_) in tumor, spleen, and lymph nodes, and decreased the immunosuppressive Treg cells in tumor, resulting in an excellent immunotherapy efficacy. This work demonstrates the joint use of antidepressant PX and biomimetic nanoparticles to promote T cell circulation and antitumor ability to enhance GBM immunotherapy for the first time. The innovative use of old drug with multifunctional nanoparticles would attract considerable interests in immunotherapy of “cold” tumors including GBM or other immunosuppressive tumors.

## 4 Experimental Section

### Materials

CuCl_2_⋅2H_2_O (≥99%), Se powder (≥99.5%), sodium borohydride (NaBH_4_, 99%), mercaptosuccinic acid (MSA, 99%) were purchased from Sigma‐Aldrich. *β*‐cyclodextrin (CD) was purchased from Shangdong Binzhou Zhiyuan Biotechnology Co. Ltd. JQ1 was purchased from Shanghai Selleck Chemicals Co. Ltd. DSPE‐PEG‐CD6 was purchased from Xian Ruixi Biotechnology Co. Ltd. 2,7‐dichlorofluorescein diacetate (DCFH‐DA) was purchased from Thermo Fisher Scientific. Milli‐Q water (>18 MΩ·cm) was used in the experiments. All chemicals and reagents were used as received without any further purification.

GL261 cells and GL261‐Fluc cells expressing firefly luciferase were obtained from Nanjing Kebai Biotechnology Co. Ltd. Dulbecco's modified Eagle's medium (DMEM) media, fetal bovine serum (FBS), penicillin, and streptomycin (Pen Strep) were bought from LANSO Biotechnology Co. Ltd. Antibody‐mediated depletion/neutralization experiments, mice were pretreated with anti‐CD3 (Biolegend, cat. no. 100236), anti‐CD8 (Biolegend, cat. no. 100708), anti‐CD4 (Biolegend, cat. no. 100510), anti‐PD‐1 (Biolegend, cat. no. 135205), anti‐TIM‐3 (Biolegend, cat. no. 134007), anti‐PD‐L1 (Biolegend, cat. no. 135206), anti‐CD16/32 (Biolegend, cat. no. 156604), anti‐CD11c (Biolegend, cat. no. 117308), anti‐CD80 (Biolegend, cat. no. 104714), anti‐CD3 (Biolegend, cat. no. 100236), anti‐CD86 (Biolegend, cat. no. 105032), anti‐CD25 (Biolegend, cat. no. 102008), anti‐CD44 (Biolegend, cat. no. 103044), anti‐CD62L (Biolegend, cat. no. 104436), anti‐S1PR1 (Thermo Fisher, MA5‐32587), anti‐GRK2 (Abcam, ab228705).

### Characterization

The morphology of nanoparticles was characterized by TEM (FEI Tecnai F20) operating at an acceleration voltage of 200 kV. The UV‐vis–NIR absorbance was recorded with a PerkinElmer Lambda 750 UV‐vis‐NIR spectrophotometer. The flow cytometry was characterized by FACMVerse (BD). The fluorescence of cells and tissues was analyzed by a scanning confocal microscope (FV1200, Olympus).

### Synthesis of CS‐J@CM NPs

CS‐J NPs were synthesized as in previous work.^[^ ^43^ ^]^ To improve the homologous adhesion of nanoparticles, GL261 cell membranes modified with CD6 were used to coat the CS‐J NPs (the ratio of CS‐J and cell membrane was 10:1) to result in CS‐J@CM/6 NPs. GL261 cells were first harvested and washed with phosphate‐buffered saline (PBS) three times and resuspended in a cold Tris buffer (pH = 7.4) (the buffer solution was consisted of 10 × 10^−3^ m MgCl_2_, 10 × 10^−3^ m Tris, and 1×ethylenediamine tetra‐acetic acid (EDTA)‐free protease inhibitor) at 4 °C for 1 h, before being sonicated for 10 min in an ultrasonicator at 0 °C. The solution was then centrifuged at 600 rpm for 10 min at 4 °C to remove cell nucleus, and the resultant supernatants were centrifuged at 11 480 rpm for 10 min to separate other organelle. The obtained supernatants were further centrifuged at 110 000 rpm for 30 min to collect cell membranes, which were resuspended and extruded through 400 nm polycarbonate membranes for five cycles. The GL261 cell membranes were mixed with DSPE‐PEG‐CD6 under ultrasonication for 20 min to form CM‐CD6 hybrid membrane (referred as CM/6). Afterward, the mixture of CS‐J NPs solution and cell membranes was extruded through 200 nm polycarbonate membranes for at least five cycles. The resultant sample was denoted as CS‐J@CM/6 NPs.

### Fenton‐Like Reaction of CS‐J@CM/6 NPs

The degradation of H_2_O_2_ by CS‐J@CM/6 NPs was characterized by an indicator containing 24% Ti(SO_4_)_2_ (1.33 mL) and 8.33 mL H_2_SO_4_ in 50 mL H_2_O. H_2_O_2_ (400 × 10^−6^ m ) was mixed with CS‐J@CM/6 NPs (12.5 µg mL^−1^) in 1 mL H_2_O. The residual H_2_O_2_ was detected by measuring the absorbance of reaction solution at 405 nm. The generation of total ROS was measured by the fluorescence of dichlorofluorescein diacetate (DCFH‐DA). The generated O_2_ was characterized with a dissolve oxygen meter (JPBJ‐608).

### In Vitro Assessment of CS‐J@CM NPs Crossing the BBB

The in vitro BBB model was established by using a transwell system. The bEnd.3 cells were seeded on the inset transwell plates with the polyester membrane (1 × 10^5^ cells per well). The GL261 cells were cultured in the FBS‐free DMEM medium in basolateral. When the TEER values reached to 150 Ω·cm^2^, the CS‐J, CS‐J@CM, or CS‐J@CM/6 NPs were then added in the upper chambers. After 4 h incubation, the TEER value of cells was analyzed. In addition, the GL261 cells in basolateral were harvested and digested with HNO_3_/H_2_O_2_ (2:1 = V_1_/V_2_). The copper concentrations in the cells were measured by ICP‐MS.

### In Vitro Cytotoxicity

GL261 cells were seeded in the 96‐well plates with a density of 8 × 10^3^ to 1 × 10^4^ cells per well for 24 h, and different concentrations of CS‐J@CM/6 NPs dispersed in DMEM (0.8, 1.6, 3.2, 6.2, 12.5, 25 µg mL^−1^) were added. After cultured for 4 h, the medium was removed and washed twice by PBS, and then the cytotoxicity of CS‐J@CM/6 NPs was characterized by the standard MTT assay.

### PD‐L1 Expression by GL261 Cells after Cultured with CS‐J@CM/6 NPs

The PD‐L1 expressed by GL261 cells was detected as described elsewhere.^[^ ^43^ ^]^

### S1PR1 Expression by T Cells Extracted from BM of GBM‐Bearing Mice and Cultured with CS‐J@CM/6 NPs

The CD3^+^ T cells were extracted from BM of GBM‐bearing mice using the MojoSort Mouse CD3^+^ T cells isolation Kid (B306673) and seeded in the 12‐well plates (1 × 10^6^ cells per well) and incubated with or without PX, JQ1, or CS‐J@CM/6 NPs (12.5 µg mL^−1^) in the presence of IL‐2 at 37 °C under 5% CO_2_ for 12 h. The cells were then washed twice with PBS and re‐dispersed in 100 µL anti‐S1PR1‐APC for 20 min on ice. Afterward, the cells were washed twice with PBS, and then analyzed with flow cytometry analysis.

### Immunogenic and DC Maturation Induced by CS‐J@CM/6 NPs

The DC maturation was characterized by the similar method reported in previous work.^[^ ^43^ ^]^

### PD‐1/TIM‐3 Expression by T Cells Extracted from the Spleen of C57BL/6 Mice and Cultured with CS‐J@CM/6 NPs

The T cells were extracted from the spleen of GBM‐bearing mice using MojoSort Mouse CD3^+^ T cells isolation Kid (B306673), seeded in the 12‐well plates (1 × 10^6^ cells per well) and incubated with matured DCs in the presence of IL‐2 for 24 h, followed by incubation with CS‐J NPs or CS‐J@CM/6 NPs for 12 h. The cells were washed twice with PBS and then re‐dispersed in 100 µL of the mixture solution containing anti‐PD‐1‐PE or anti‐TIM‐3‐APC on ice. The cells were washed twice with PBS, and then analyzed with flow cytometry.

### Orthotopic Glioblastoma Model

All animal experiments were carried out according to the guidelines approved by the ethics committee of Soochow University (Soochow, China). Male C57BL/6 mice, aged 8 weeks, were supplied by laboratory animal center of Soochow University.

To create an orthotopic glioblastoma model, a solution of GL261 cells (3 × 10^5^) in PBS (5 µL) was injected into the mice's striatum (bregma was 1.0 mm, right lateral was 2.0 mm), and the depth was 2.5 mm. The orthotopic malignant glioblastoma‐bearing mice were treated with different therapy methods after tumor cells were inoculated for 6 days.

### PX Treatment Studies

The orthotopic glioblastoma mice were orally administrated with PX (10 mg kg^−1^ every 2 days) for four times. The expression of GRK2 or S1PR1 on T cells was obtained from the BM after 3 days treatment and analyzed by Western blot analysis and flow cytometry.

### Western Blot Experiment

The GL261 cells were seeded in the 12‐well plates (8 × 10^4^ to 1 × 10^5^) and incubated for 24 h. The cells were then cultured with JQ1, CS‐J NPs, or CS‐J@CM/6 NPs (12.5 µg mL^−1^) at 37 °C under 5% CO_2_ for 4 h, washed twice with PBS, and total proteins were collected by lysing cells with radio immunoprecipitation assay buffer (Beyotime, P0013B) containing protease inhibition cocktails. The protein concentration was determined by the enhanced BCA Protein Assay Kit (Beyotime, P0010). Samples containing 30 µg of total cell proteins were separated by sodium dodecyl sulfate‐polyacrylamide gel electrophoresis gels (8%, 10%, or 12%), and then transferred onto a polyvinylidene fluoride membrane (0.45 µm in aperture). The membrane was blocked with 5% milk in Tris‐Buffer Saline Tween‐20 (TBST) solution containing 0.1% Tween‐20 for 2 h at room temperature, and then incubated overnight at 4 °C with one of the following primary antibodies, which were used according to the instructions of the manufacturer. The antibodies were diluted as follows: anti‐Calreticulin (Abcam, ab92516), anti‐HMGB1 (Abcam, ab79823), anti‐S1PR1 (Abcam, ab11424), anti‐GRK2 (Cell Signaling Technology), anti‐PD‐L1 (Abcam, ab213480). After washed by TBST solution three times, the membrane was continued to be incubated for 1 h at room temperature by one of the secondary antibodies, which was selected according to the primary antibody. The secondary antibodies were goat anti‐rabbit IgG‐HRP (Beyotime, A0208) and goat anti‐mouse IgG‐HRP (Beyotime, A0258). The HPR on the secondary antibodies reacted with BeyoECL Star detection reagent (Beyotime, P0018AS), and emitted fluorescence under excitation, which was recorded by FluorChem M (Alpha). The quantification of Western blot results was analyzed by using Image J software.

### Immunofluorescence Staining

GL261 cells were seeded in the 6‐well plates, and then incubated with JQ1, CS‐J NPs, or CS‐J@CM/6 NPs (12.5 µg mL^−1^) at 37 °C under 5% CO_2_ for 4 h, washed twice with PBS. Then the cells were collected for further immunofluorescent staining analysis. The cells were fixed with 4% paraformaldehyde for 30 min, exposed to a membrane breaking fluid (1% Triton X‐100) for 30 min, washed with PBS three times (5 min each time), and followed by blocking with 5% bovine serum albumin solution at 37 °C for 1 h. After that, they were incubated with specific diluted primary antibodies overnight at 4 °C, including anti‐Calreticulin (Abcam, ab92516) or anti‐PD‐L1 (Abcam, ab213480). Then, the cells were washed with PBS three times (5 min each time) and stained with the corresponding diluted fluorescent dye‐linked secondary antibody at 37 °C for 1 h without light, and then washed with PBS three times (5 min each time), and followed by staining nucleus with Hoechst 33342 dye for 10 min. Finally, after the cells were washed with PBS three times, their fluorescence images were observed by using Confocal laser scanning microscopy (CLSM, FV1200, Olympus, Japan).

### In Vivo Immunotherapy of Orthotopic Glioblastoma

The GL261 mice bearing orthotopic glioblastoma were classified randomly into four groups, in which each group had ten mice. The four groups were 1) Control group, 2) PX group, 3) PX + CS‐J group, and 4) PX + CS‐J@CM/6 group, respectively. The injection dose of CS‐J@CM NPs was 5 mg kg^−1^ for each mouse. Their brains were also collected for H&E staining to examine the antitumor efficacy.

### In Vivo Monitoring of Tumor Growth and Survival Rates

A solution of GL261‐Luciferase cells (5 × 10^5^) in PBS (5 µL) was injected into the mice's striatum (bregma was 1.0 mm, right lateral was 2.0 mm), and the depth was 2.5 mm. The orthotopic glioblastoma bearing mice were treated with different therapeutic methods after tumor cells were inoculated for 6 days. The tumor growth was monitored by measuring bioluminescence through the IVIS Lumina XRMS Series Imaging System. In addition, the survival rates were recorded and analyzed by GraphPad (Prism 8.0).

### Biodistribution of CS‐J@CM/6 NPs

GL261 tumor‐bearing mice were intravenously injected with 200 µL of CS‐J@CM/6 NPs solution. They were divided into six groups. Their various organs or tissues, such as heart, liver, spleen, lung, kidney, blood, and tumor, were resected from the mice in each group at 1, 3, 5, 7, and 15 days postinjection, and then dissolved in the digesting solution HNO_3_/H_2_O_2_ (2:1 = V_1_/V_2_). The copper concentrations in each organ/tumor were determined by ICP‐MS.

### TUNEL Staining

The GL261 tumor‐bearing mice were injected with a solution of PBS or CS‐J@CM/6 NPs (dose: 5 mg kg^−1^) via their tail veins. After twice postinjection, the tumors from four groups of mice were harvested and stained with TUNEL, and examined with CLSM.

### Flow Cytometry

The treated tumor‐bearing mice were sacrificed and their CLNs and spleen were harvested after different treatments. The cells were isolated by Collagenase Type I (1 mg mL^−1^, purchased from Gibico) for 1 h at 37 °C. After washed with PBS twice, the cell pellets were suspended in 3 mL of Ack lysis buffer for lysing red blood cells at 4 °C for 5 min. After centrifugation for 5 min (1000 rpm), the single cell suspension was washed again by PBS for twice. Then the CD16/32 was used to block nonspecific binding sites at 4 °C for 10 min. The cells were detected by flow cytometry analysis: 1) CD8^+^ T cells (CD3^+^CD8^+^), 2) Tregs (CD3^+^CD4^+^CD25^+^Foxp3^+^), 3) T_EM_ (CD3^+^CD44^+^CD62L^low^), 4) DCs (CD11c^+^CD80^+^CD86^+^).

### Statistical Analysis

The data were expressed as mean ± SD using GraphPad (Prism 8.0). One‐way analysis of variance statistical was used to calculate the experimental data. The data were classified by the values of *p* and denoted by (*) for *P* < 0.05, (**) for *P* < 0.01, (***) for *P* < 0.001.

## Conflict of Interest

The authors declare no conflict of interest.

## Supporting information

Supporting Information

## Data Availability

The data that support the findings of this study are available from the corresponding author upon reasonable request.
