# Supplementary material for: Reversing T Cell Dysfunction to Boost Glioblastoma Immunotherapy by Paroxetine‐Mediated GRK2 Inhibition and Blockade of Multiple Checkpoints through Biomimetic Nanoparticles
Source: Adv Sci (Weinh). 2023 Jan 25;10(9):2204961. doi: 10.1002/advs.202204961 (PMC10037995; doi:10.1002/advs.202204961)
Supplement: Supplementary file 1 — Supporting Information [file ADVS-10-2204961-s001.pdf]

Supporting Information

Reversing T Cell Dysfunction to Boost Glioblastoma Immunotherapy by Paroxetine-mediated GRK2 Inhibition and Blockade of Multiple Checkpoints through Biomimetic Nanoparticles

*Tingting Wang, Hao Zhang, Yaobao Han, Qing Zheng, Hanghang Liu, Mengxiao Han, Zhen Li**

Center for Molecular Imaging and Nuclear Medicine, State Key Laboratory of Radiation Medicine and Protection, School for Radiological and Interdisciplinary Sciences (RAD-X), Suzhou Medical College of Soochow University, Collaborative Innovation Center of Radiation Medicine of Jiangsu Higher Education Institutions Suzhou 215123, China
Email: zhenli@suda.edu.cn

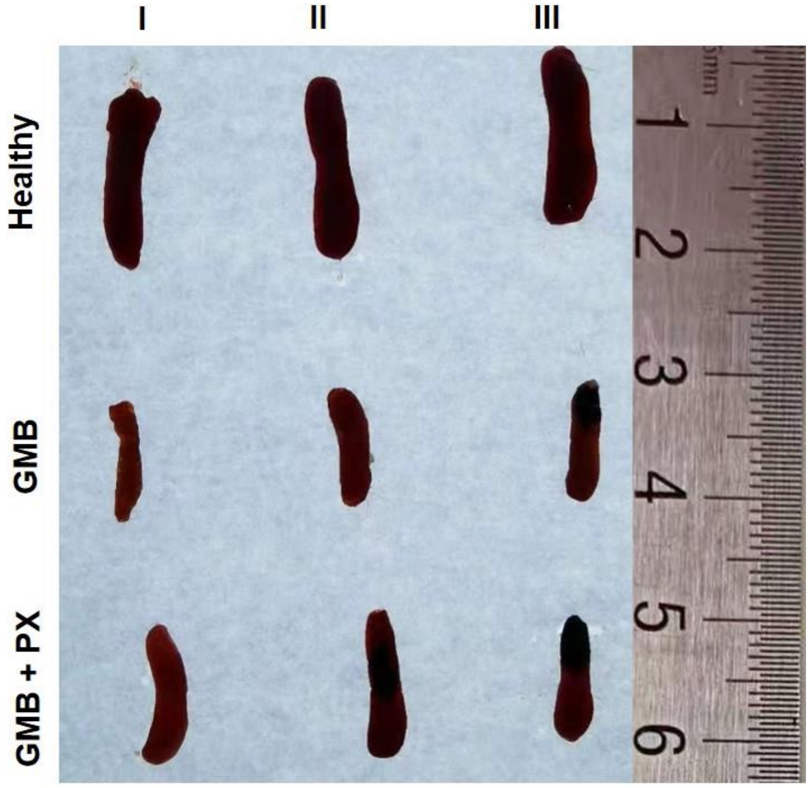

Figure S1. The image of spleens taken from healthy C57BL/6 mice, orthotopic GBM-bearing mice, and the GBM-bearing mice treated with PX.

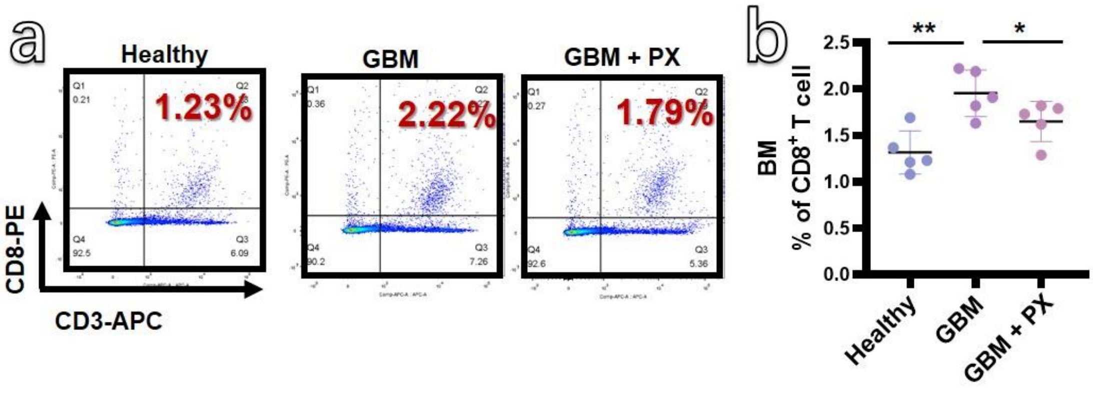

Figure S2. (a) Flow cytometry analysis of $CD8^+$ T cells ($CD3^+CD8^+$) in the BM of mice from the Healthy group, GBM group, and GBM + PX group. (b) The percentage of $CD8^+$ T cells in the BM from different groups of mice (n=5).

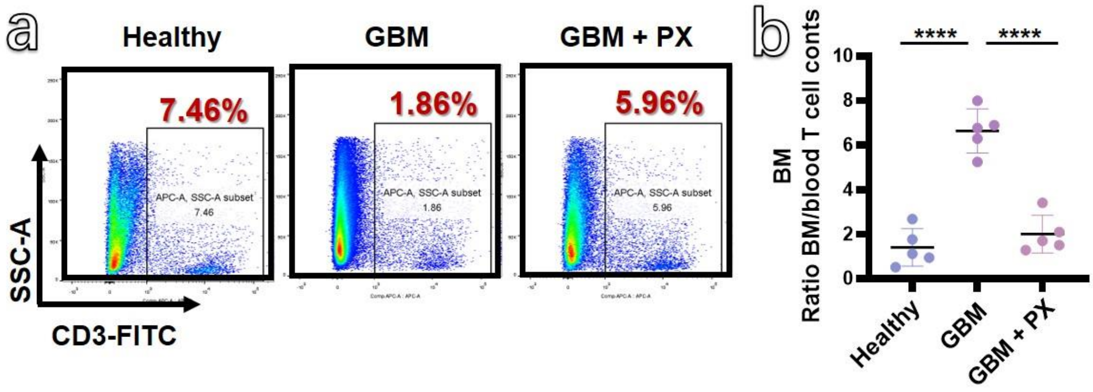

Figure S3. (a) Flow cytometry analysis of CD3$^+$ T cells in the blood of mice from the Healthy group, GBM group, and GBM + PX group. (b) The ratio of CD3$^+$ T cells in the BM and blood. Each group consisted of 5 mice (n=5).

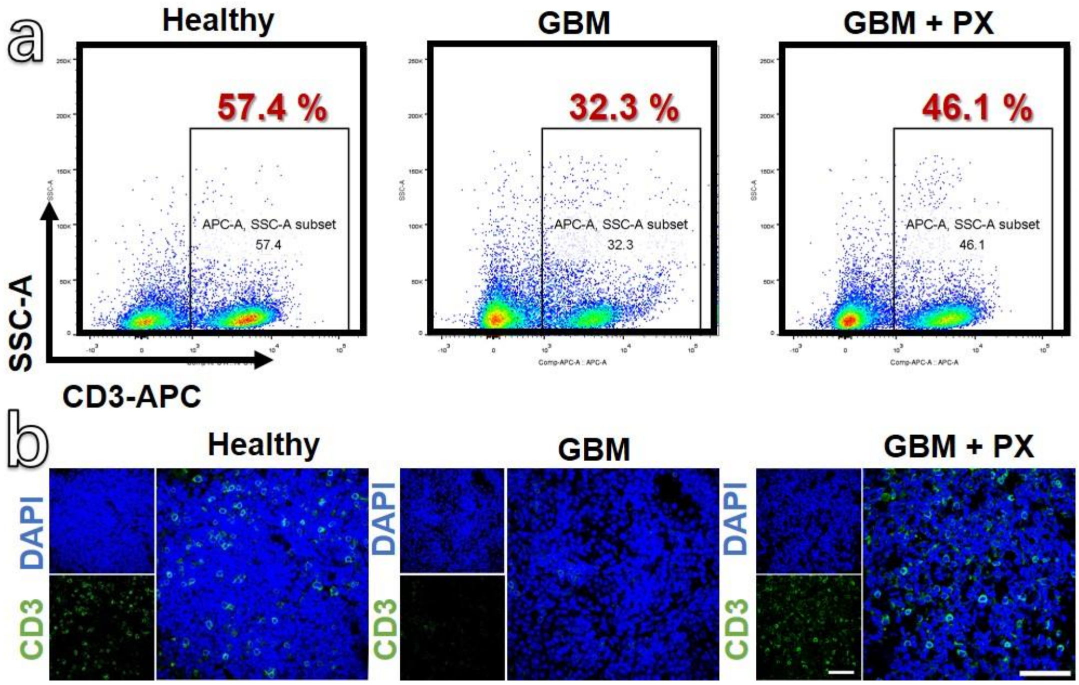

Figure S4. (a) Flow cytometry analysis of CD3$^+$ T cells in the lymph nodes of mice from the Healthy group, GBM group, and GBM + PX group. (b) Immunofluorescence images of CD8$^+$ T cells in these lymph nodes (scale bar: 100 μm).

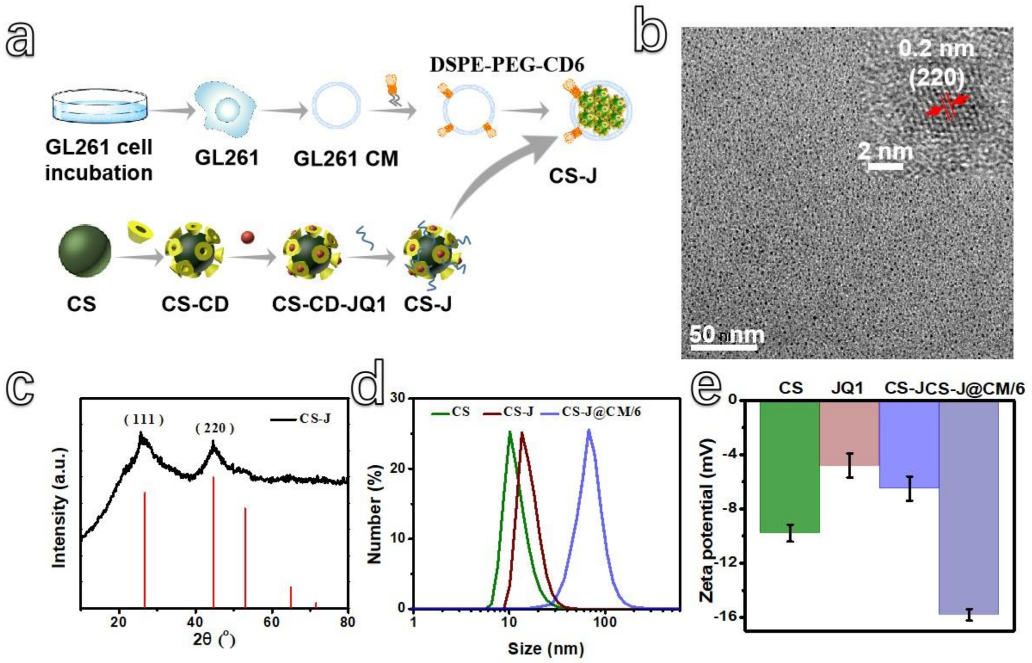

Figure S5. (a) Schematic illustration of preparation of CS-J@CM/6 NPs. (b) TEM image of CS-J NPs (scale bar: 50 nm) with an inset of high-resolution TEM image. (c) XRD pattern of ultrasmall CS-J NPs in comparison with standard peaks of cubic berzelianite (JCPDS Card No. 06-0680). (d) Hydrodynamic sizes of CS, CS-J, and CS-J@CM/6 NPs. (e) Zeta potential of CS, JQ1, CS-J, and CS-J@CM/6 NPs.

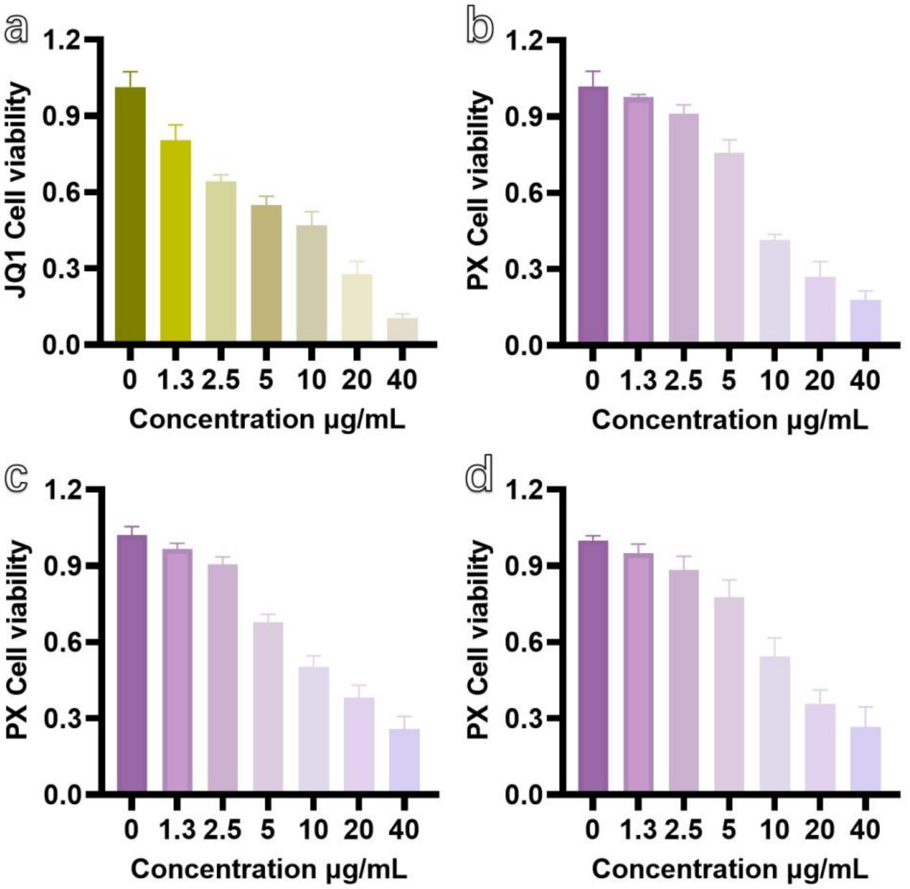

Figure S6. Viability of CD3+ T cells cultured with different concentrations of (a) JQ1 and (b) PX. (c, d) Viability of BV2 cells and SH-SY5Y cells cultured with different concentrations of PX.

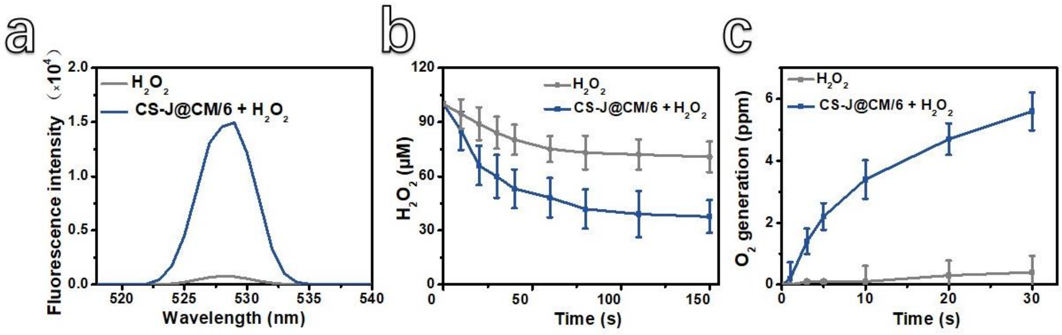

Figure S7. (a) Fluorescence spectra of 2,7-dichlorofluorescin diacetate (DCFH-DA) mixed with H_2O_2 solution or a mixture of H_2O_2 and CS-J@CM/6 NP solutions. (b) Degradation of H_2O_2 (400 μM) catalyzed by CS-J@CM/6 NPs (12.5 μg/mL). (c) Generation of O_2 from a solution of CS-J@CM/6 (12.5 μg/mL) NPs and H_2O_2 (400 μM).

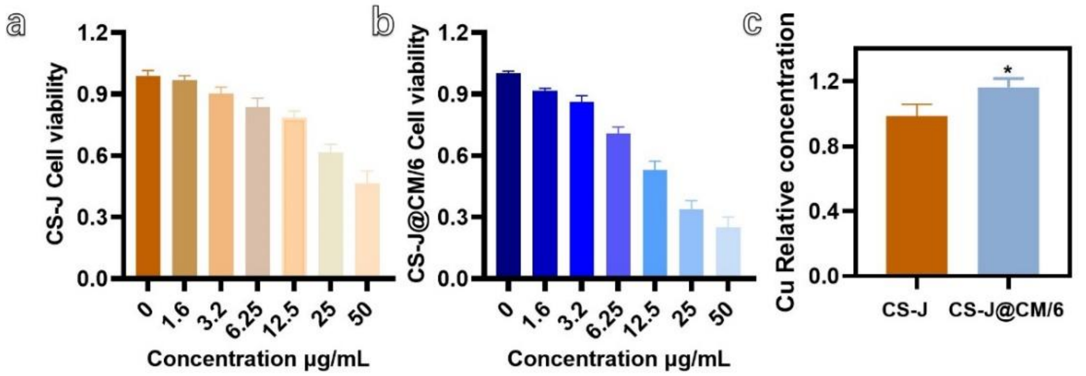

Figure S8. Viability of GL261 cells cultured with different concentrations of (a) CS-J NPs and (b) CS-J@CM/6 NPs. (c) The Cu concentration in GL261 cells detected by ICP-MS.

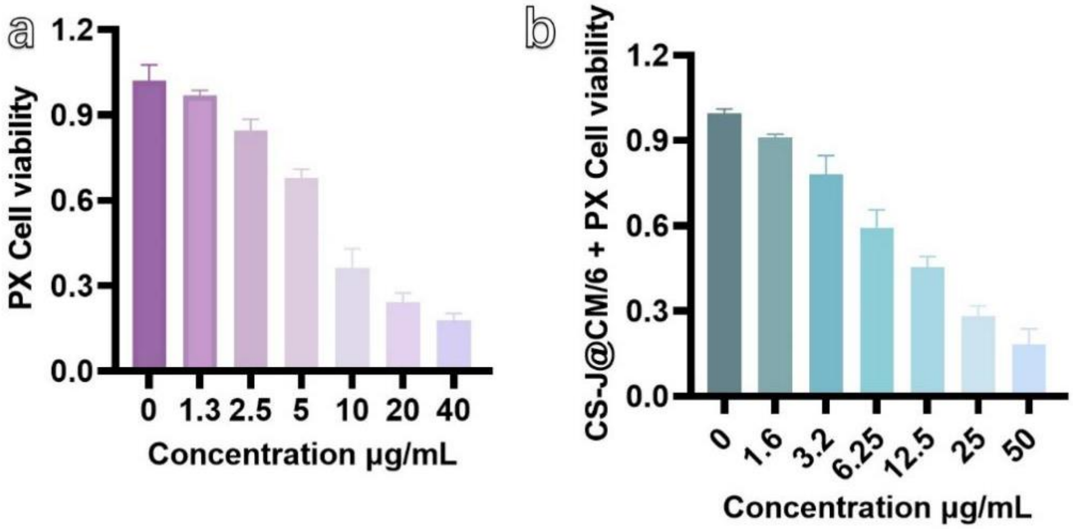

Figure S9. Viability of GL261 cells cultured with different concentrations of (a) PX, and (b) PX (5 μg/mL) mixed with different concentrations of CS-J@CM/6 NPs.

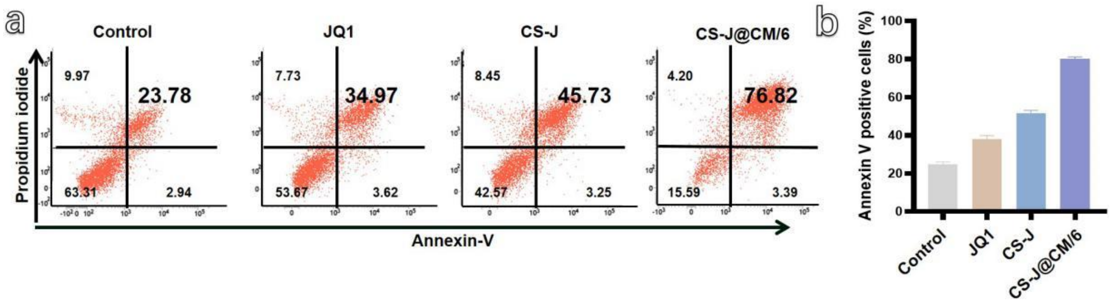

Figure S10. (a) Apoptotic cell ratios determined by flow cytometry. (b) Corresponding statistical percentage of apoptotic GL261 cells (n = 3).

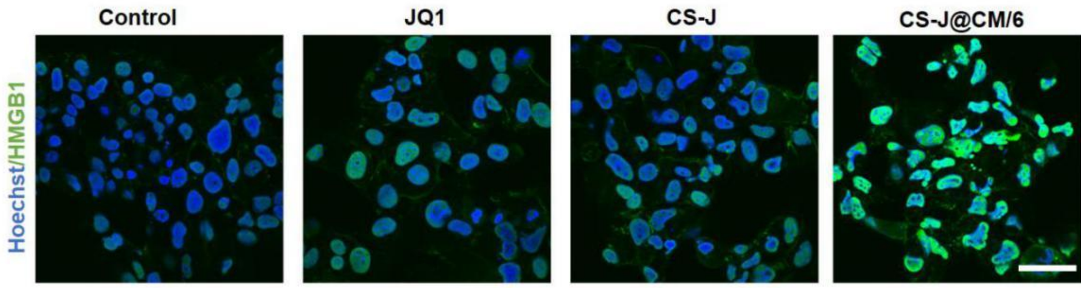

Figure S11. Immunofluorescence images of HGBM released by GL261 cells (scale bar: 50 μm).

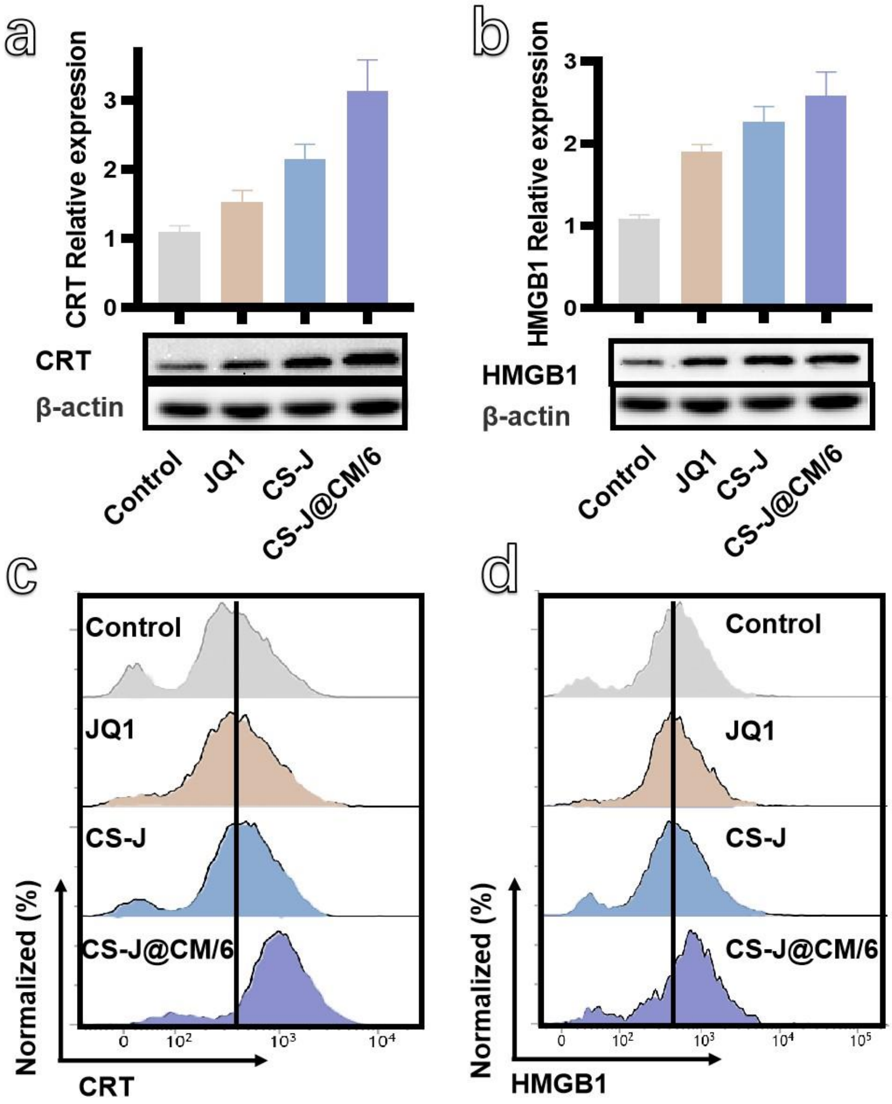

Figure S12. (a, c) Detection of CRT exposure on the membrane of GL261 cells from different groups by Western blot and flow cytometry. (b, d) Detection of HMGB1 released by GL261 cells from different groups by Western blot and flow cytometry.

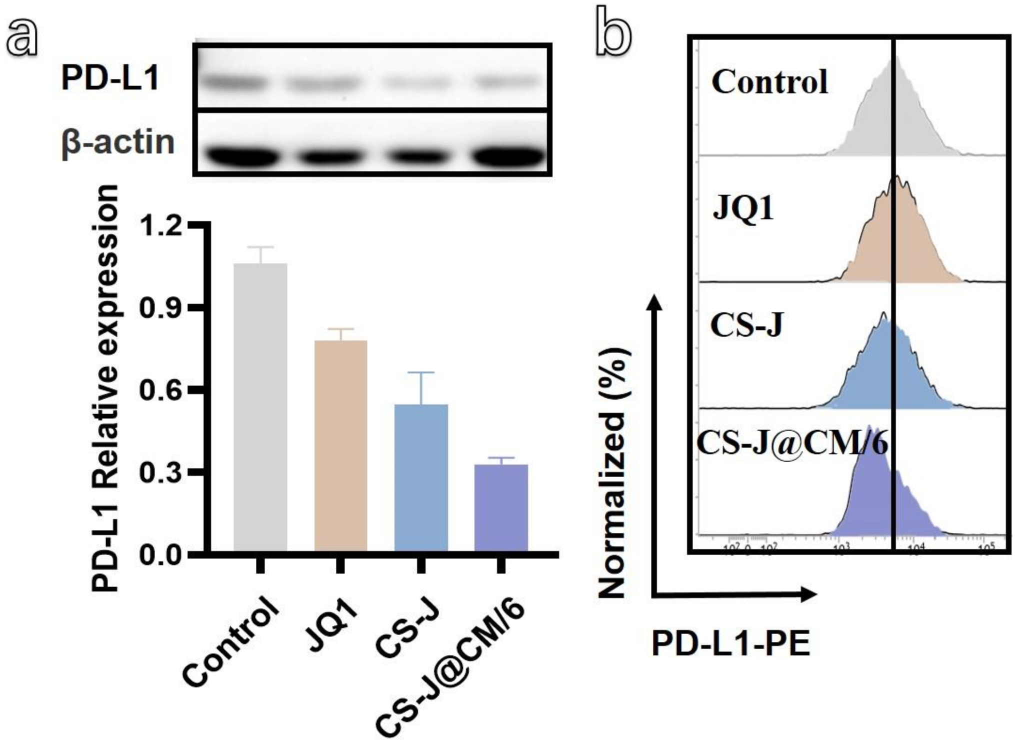

Figure S13. (a) Detection of PD-L1 expression by the GL261 cells from different groups by Western blot. (b) Flow cytometry analysis of PD-L1 expression (n=3) .

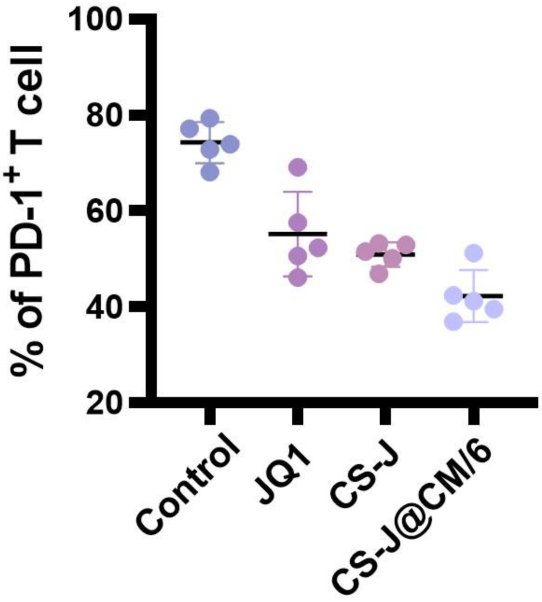

Figure S14. The percentage of PD-1^{+}CD3^{+} T cells after CD3^{+} T cells were incubated with JQ1, CS-J or CS-J@CM/6 (n=5).

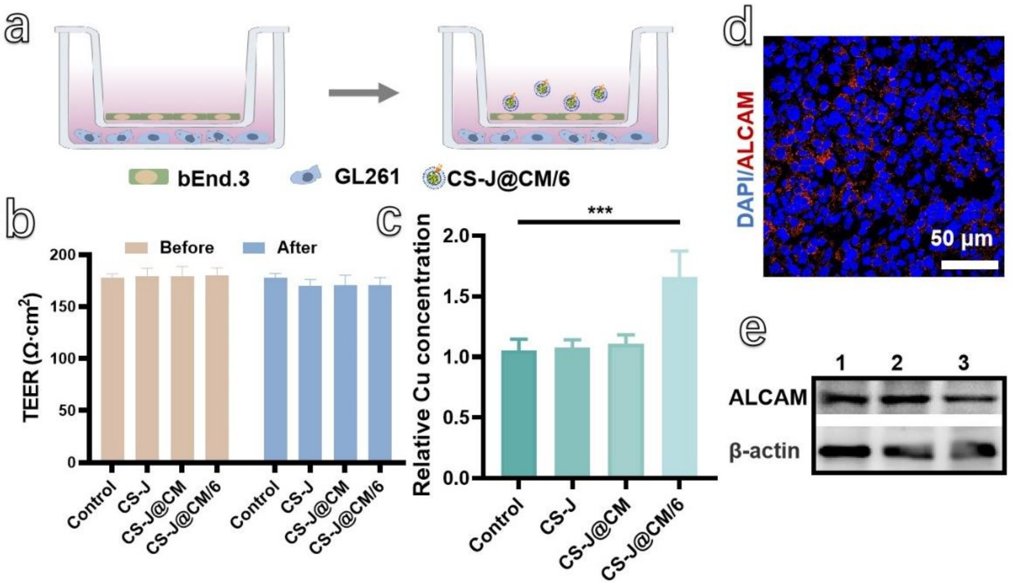

Figure S15. Characterization of targeting capability of CS-J@CM/6 NPs to the GL261 cells. (a) Schematic diagram of the setup of *in vitro* BBB transwell model. (b) Transepithelial electrical resistance (TEER) values of monolayered bEnd.3 cells before and after treatment with CS-J, CS-J@CM or CS-J@CM/6 NPs. (c) The Cu concentration in the GL261 cells from the lower chamber detected by ICP-MS. (d) Immunofluorescence images of ALCAM in the brain tissue from the GBM mice (scale bar: 50 μm). (e) Detection of ALCAM expression in the brain tissues from the GBM mice by Western blot.

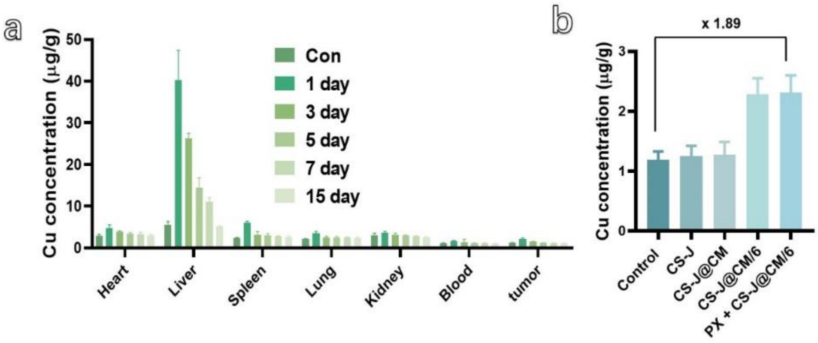

Figure S16 (a) The biodistribution of CS-J@CM/6 NPs after intravenous injection into GBM-bearing mice, as determined by measuring Cu concentrations in the tissue lysates with ICP-MS. (b) The Cu concentration in tumor from different groups of mice.

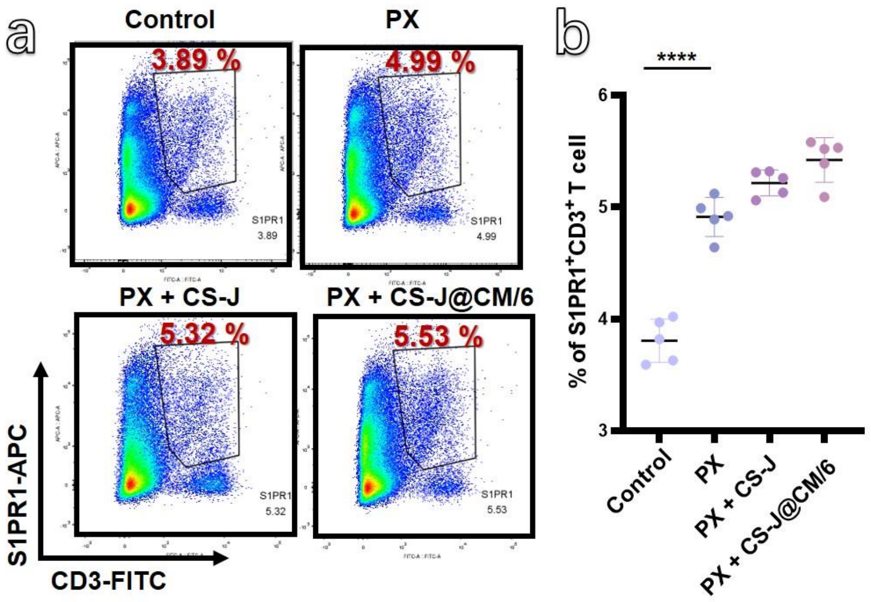

Figure S17. (a) Flow cytometry analysis of S1PR1$^+$CD3$^+$ T cells in the BM of mice from Control group, PX group, PX + CS-J group, and PX + CS-J@CM/6 group. (b) The percentage of S1PR1$^+$CD3$^+$T cells in their BM (n=5).

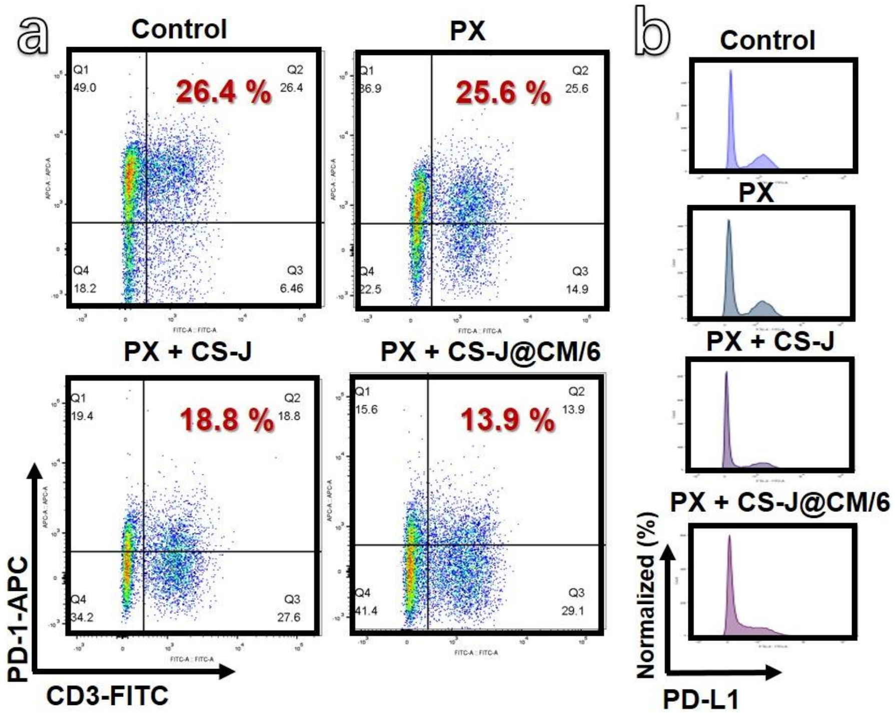

Figure S18. (a) Flow cytometry analysis of PD-1$^+$CD3$^+$T cells in the tumor from Control group, PX group, PX + CS-J group, and PX + CS-J@CM/6 group. (b) Flow cytometry analysis of PD-L1 cells in tumor from these groups.

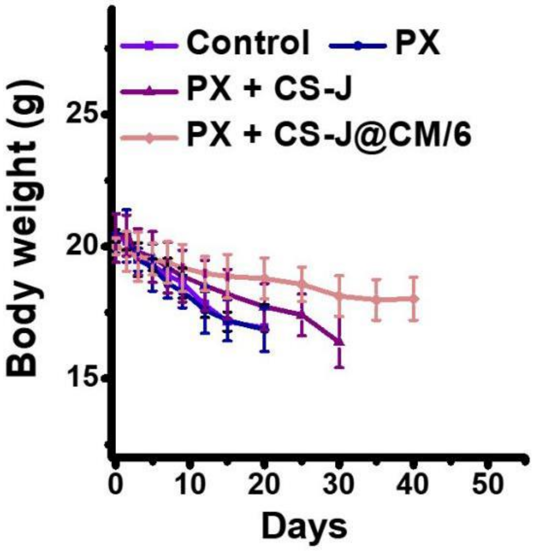

Figure S19. Weights of mice from different groups after received different treatments (n=5).

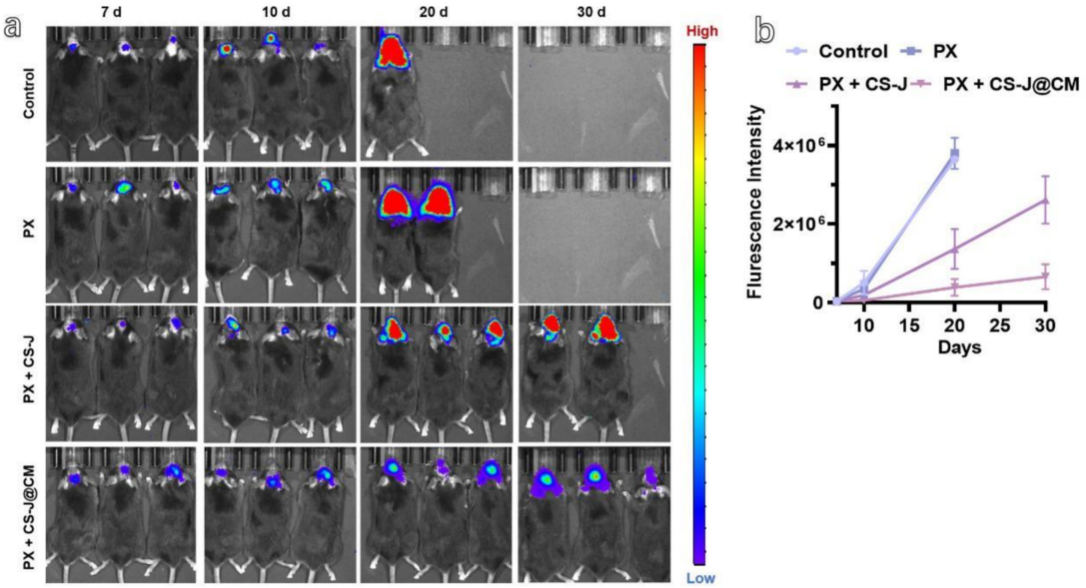

Figure S20. (a) Bioluminescence images of mice from Control group, PX group, PX + CS-J group, and PX + CS-J@CM/6 group (n=5). (b) The quantification of bioluminescence intensity from different groups of mice.

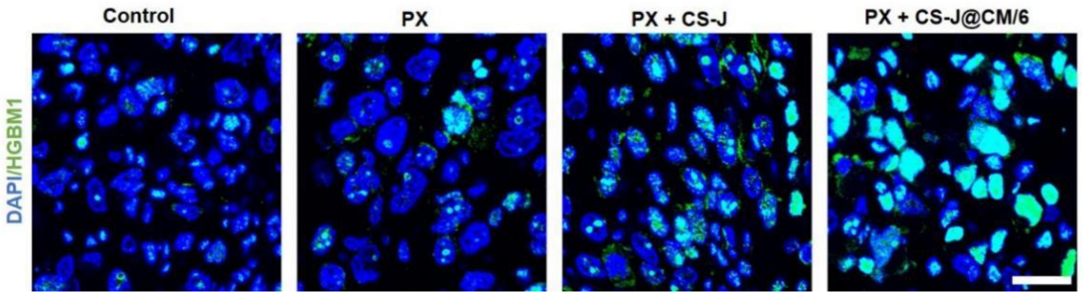

Figure S21. Immunofluorescence images of HGBM1 in tumor from different groups of mice (scale bar: 25 μm).

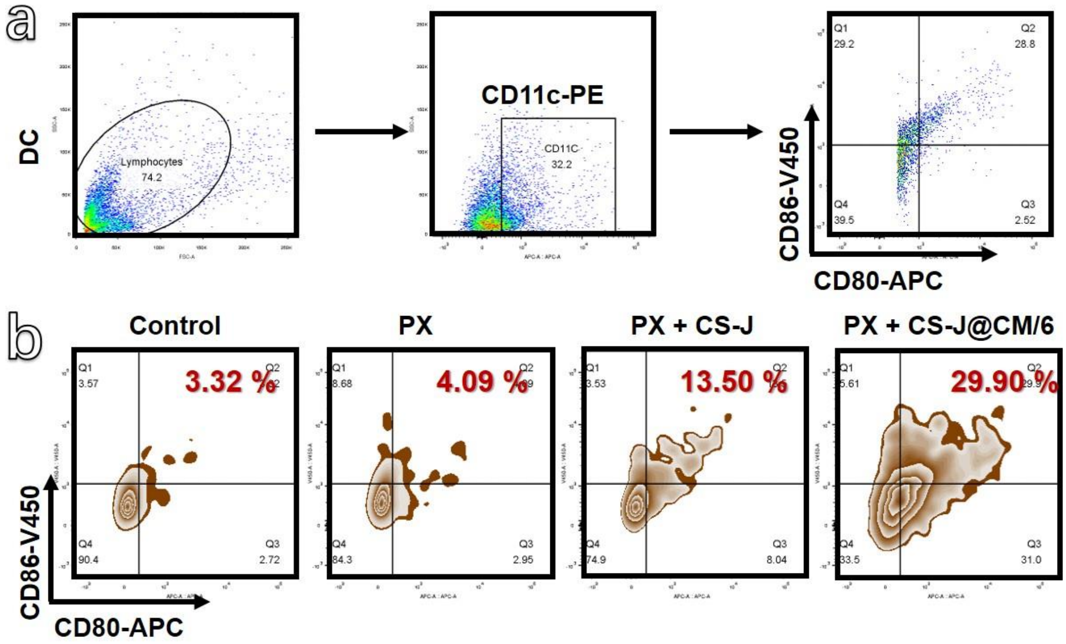

Figure S22. (a) Flow cytometry gating strategy for matured DCs. (b) Flow cytometry analysis of matured DCs ($CD11c^{+}CD80^{+}CD86^{+}$) in tumor from different groups of mice.

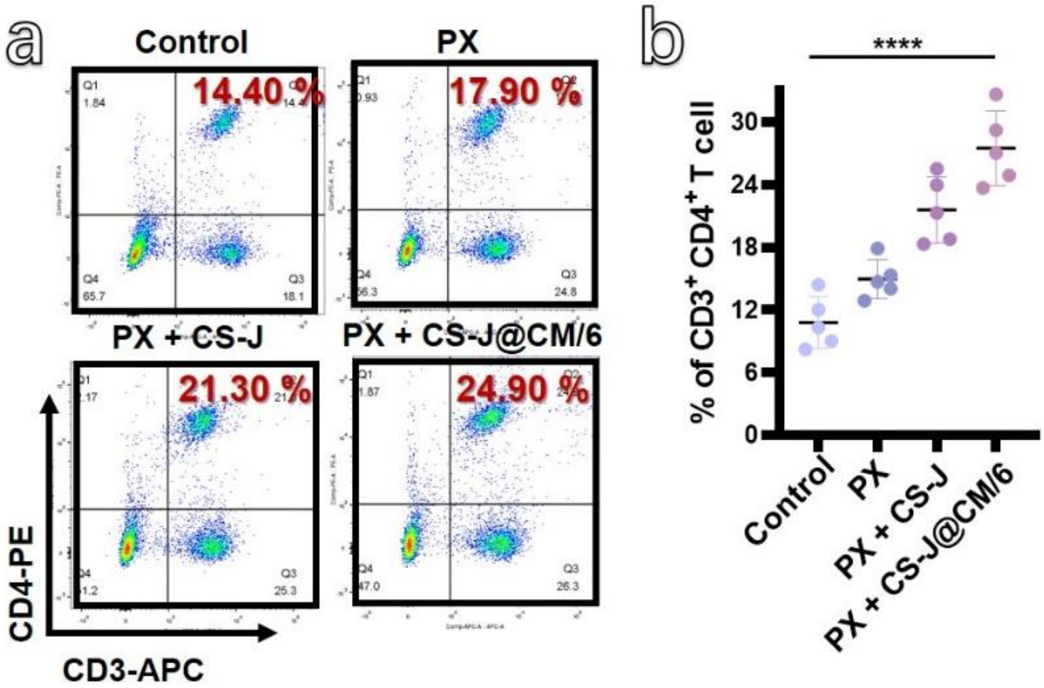

Figure S23. (a) Flow cytometry analysis of CD3$^+$CD4$^+$ T cells in the spleen of mice from Control group, PX group, PX + CS-J group, and PX + CS-J@CM/6 group. (b) The percentage of CD3$^+$CD4$^+$ T cells in the spleen of mice from different groups (n=5).

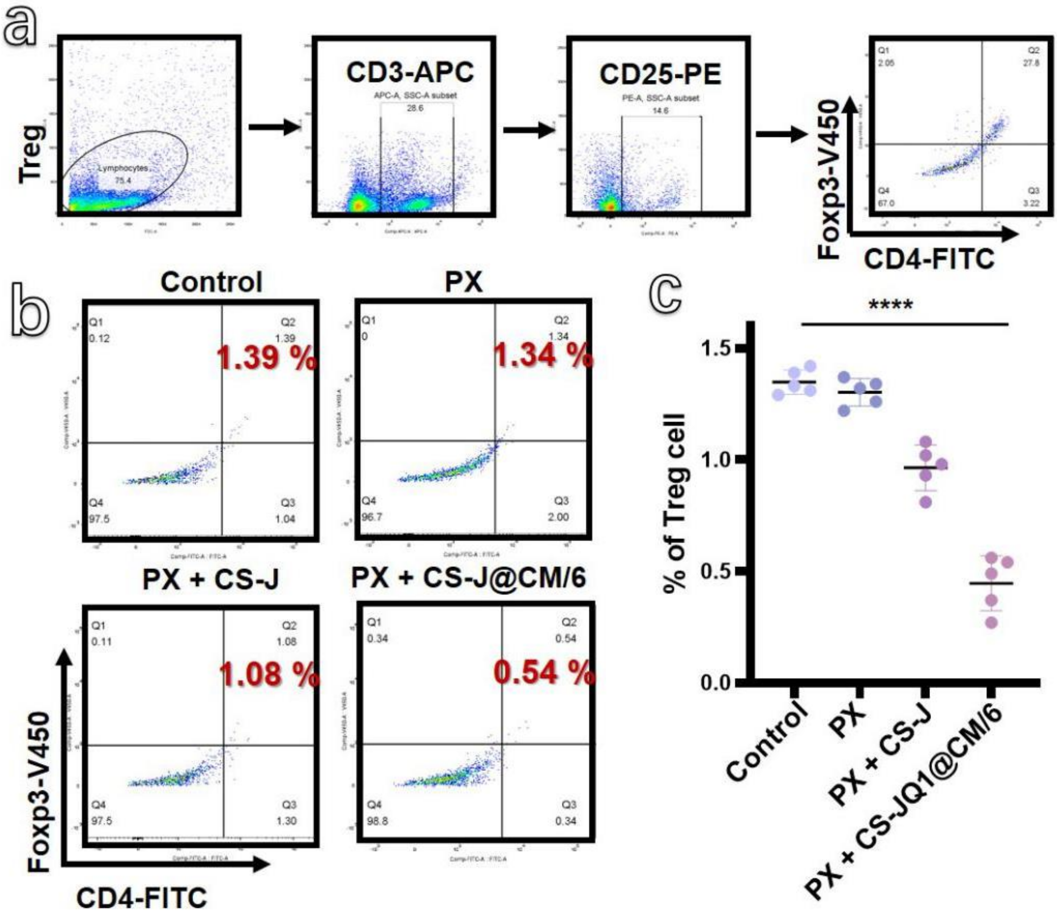

Figure S24. (a) Flow cytometry gating strategy for Treg cells. (b) Flow cytometry analysis of Treg cells in the lymph nodes of mice from Control group, PX group, PX + CS-J group, and PX + CS-J@CM/6 group. (c) The percentage of Treg cells in the lymph nodes from four groups of mice (n=5).

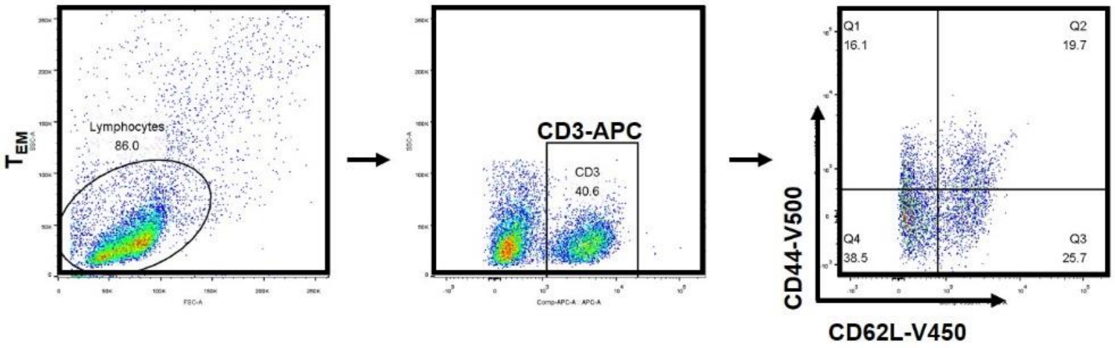

Figure S25. Flow cytometry gating strategy for T_{EM} cells.

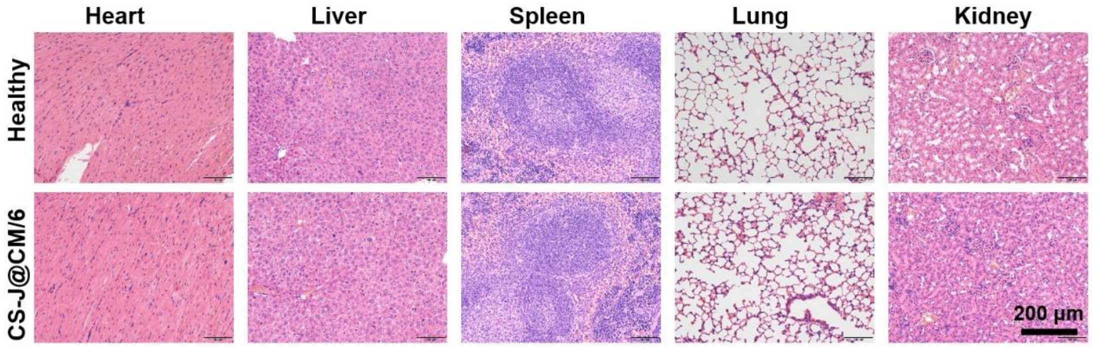

Figure S26. The H&E staining of the major organs (heart, liver, spleen, lung and kidney) collected from GBM-bearing mice at 18 days posttreatment, in comparison with those of healthy mice. There was no obvious damage to these tissues. (Scale bar: 200 μm).
